# The Relationship Between Gut Microbiome Features and Chemotherapy Response in Gastrointestinal Cancer

**DOI:** 10.3389/fonc.2021.781697

**Published:** 2021-12-23

**Authors:** Ningning Li, Chunmei Bai, Lin Zhao, Zhao Sun, Yuping Ge, Xiaoyuan Li

**Affiliations:** Department of Medical Oncology, Peking Union Medical College Hospital, Chinese Academy of Medical Sciences and Peking Union Medical College, Beijing, China

**Keywords:** gut microbiota, gastrointestinal cancer, chemotherapy, *Roseburia*, prognosis

## Abstract

**Objective:**

The prognosis of advanced gastrointestinal cancer is poor. There are studies indicating that gut microbes might have the predictive ability to evaluate the outcome of cancer therapy, especially immunotherapy. There is limited evidence to date on the influence of microbes on chemotherapeutic response.

**Design:**

In total, 130 patients with advanced or metastatic esophageal (n=40), gastric (n=46), and colorectal cancer (n=44) were enrolled. We included 147 healthy people as controls and used 16S rRNA sequencing to analyze the fecal microbiota.

**Results:**

Significant differences in the abundance of fecal microbiota between patients with gastrointestinal cancer and controls were identified. The abundance of *Bacteroides fragilis*, *Escherichia coli*, *Akkermansia muciniphila*, *Clostridium hathewayi*, and *Alistipes finegoldii* were significantly increased in the patient group. *Faecalibacterium prausnitzii*, *Roseburia faecis, Clostridium clostridioforme*, *Blautia producta*, *Bifidobacterium adolescent*, and *Butyricicoccus pullicaecorum* taxa were significantly more abundant in the controls. The amount of *R. faecis* in non-responders (NR) was more likely to decrease significantly after chemotherapy, while the amount mostly increased in responders (R) (*P*=0.040). The optimal abundance variation of *R. faecis* may be a predictor for distinguishing patients with PD from those with non-PD in all patients with gastrointestinal cancer, with a sensitivity of 75.0% and a specificity of 93.9%.

**Conclusion:**

The gut microbiome of patients with esophageal cancer, gastric cancer, and colorectal cancer differs from those of healthy people. The abundance alteration of *R. faecis* in patients with GI cancer might be a predictor of chemotherapy efficacy.

## Introduction

Cancer in the gastrointestinal (GI) tract is one of the most common causes of death worldwide (1). The morbidity and mortality of esophageal cancer (EC), gastric cancer (GC), and colorectal cancer (CRC) increased during the past decade in Asia, including China (2). Accumulating evidence indicates that GI cancers developed through multiple factors; genetic alterations, immune status, diet and environmental factors, and microorganisms may be relevant (3).

The human gut encompasses 3.8×10^13^ microorganisms, which can regulate immunity and metabolism to maintain health conditions. Gut microbiota establishes an equilibrium with the host. Disturbance of this balance may result in altered microecology, which is associated with several diseases, including cancer (4). More data have demonstrated that gut microbiota affect the initiation and development of colorectal cancer (CRC) by producing short-chain fatty acids (SCFAs), affecting signal pathways or regulating immunity (5–7). The microbiota in the digestive juice and tissues of the upper digestive tract were found to be related to the pathogenesis of EC and GC (8).

Although multimodal systemic therapy has improved the outcomes of advanced GI cancers in the past half century, the unpredictable treatment response and acquired resistance are still major issues at present. To date, research on “oncomicrobiome” has been mostly focused on the role of the microbiota in the etiology of cancer (9). Some recent studies concentrated on the interaction between microbiota and immunotherapy, providing the basis for a way to improve the efficacy of immune checkpoint inhibitors and the search for novel biomarkers for efficacy prediction in cancers. Analyses using 16S ribosomal RNA (rRNA) sequencing revealed a significant enrichment in the *Ruminococcaceae* family in responsive patients with melanoma undergoing anti-programmed cell death receptor-1 (PD-1) immunotherapy (10). The *Bifidobacterium* genus was identified as a biomarker the for better efficacy of the anti-programmed cell death ligand-1 (PD-L1) inhibitor in melanoma-bearing mice (11). The role of the gut microbiota in chemotherapeutic drug modulation is equally important to immunotherapy. However, the studies on this topic were mostly basic investigations (12–14) that have indicated that chemotherapy resistance and off-target toxicity during chemotherapy may be induced by intratumor microbiota. A 2020 study with a small sample size revealed that initial 22 and 9 bacteria were treatment (chemotherapy, targeted therapy, or immunotherapy combination) responder-enriched and non-responder-enriched, respectively. However, the cohort included eight different cancer types, and only four patients had CRC (15).

Few studies to date focused on the correlation between fecal microbiota and GI tumors, particularly EC and GC in Asian populations, and had a large sample size. Characterizing the gut microbiota features in chemotherapeutic patients will address what alterations occur in the microbiota during chemotherapy regimens and may indicate the correlation with the response to treatment. To identify the specific bacteria associated with improved antitumor chemotherapy responses, we monitored the fecal bacteria of GI cancer patients over a certain period of time using the 16S rRNA miSeq Illumina platform (Illumina, Inc., San Diego, CA, USA). We tried to provide significant information on the gut microbiota in patients with GI cancer who were receiving chemotherapy and to detect the microbial differences between patients and healthy people. We aimed to identify specific bacterium related to the efficacy of chemotherapy in patients, which may support the idea that intersubject heterogeneity to chemotherapy therapeutic response could be related to the abundance variation of specific gut microbiota.

## Materials and Method

### Study Design and Sample Collection

A total of 130 patients with GI cancers who were admitted between April 2018 and April 2020 in the Department of Oncology, Peking Union Medical College Hospital (PUMCH) were enrolled. Meanwhile, 147 healthy controls (HC) were enrolled in the study during the same period. The recruited patients were histopathologically diagnosed with esophageal squamous cell carcinoma, GC, or colorectal adenocarcinoma. All of the patients were confirmed as having locally advanced or stage IV disease according to the TNM classification (American Joint Committee on Cancer 7.0) at the time of admission. The following patients were excluded: those who received any antitumor therapy within the 6 mo before admission and/or those with factors known to affect gut microbiota, such as inflammatory bowel disease, intestinal infection, bowel obstruction, and/or a history of probiotic or antibiotic usage within the 2 mo before sample collection. The HCs, aged 18–75 y, reported no history of cancer or of other diseases (GI diseases, immune, and endocrine diseases), and no history of probiotic or antibiotic usage within the 2 mo prior to sample collection. The entire study population was from northern China and had a parallel dietary background.

All of the patients received standard treatment, including chemotherapy, target therapy, or immunotherapy, according to the physicians’ choice. Clinical response was evaluated at 6–8 wk after treatment based on the response evaluation criteria in solid tumors (RECIST 1.1). All of the participants provided written informed consent prior to their enrollment in this study. This study was approved by the ethics committee of PUMCH.

The participants’ clinical data were collected from their medical records. The patients with cancer were tested for their whole blood cell count, lymphocyte subgroups, erythrocyte sedimentation rate (ESR), and hypersensitive C-reactive protein (hsCRP) before they were given antitumor treatment. Fecal samples of the patients were self-sampled at two points: 1 wk prior to the start of antitumor therapy and the time of assessment of the therapeutic effect. Fecal samples of the HCs were obtained at the Physical Examination Center of PUMCH. In total, 50 to 100 mg fecal samples were collected in a stool collection tube (OMIgene•GUT; DNA Genotek Inc., ON, Canada). The specimens were immediately stored in −80°C until further processing and testing.

### DNA Extraction and 16S rRNA Sequencing

Bacterial genomic DNA was extracted using NucleoSpin Soil DNA Kit (Macherey - Nagel Vertrieb Gmbh & Co. Kg., Düren, Germany). The purified DNA was collected as a template for polymerase chain reaction (PCR) amplification using the primers specific to the V4 variable region (515F: GTGCCAGCMGCCGCGGTAA; 806R: GGACTACHVGGGTWTCTAAT) (16) of the 16S rRNA gene. The purified PCR products (Qiagen, Hilden, Germany) were used for library pool construction.

The qualified libraries were sequenced by a HiSeq2500 gene sequencing analysis system (Illumina) and PE250 sequencing strategy. The original sequencing data were collected for analysis.

### 16S rRNA Sequence Processing and Data Analysis

Clean data were obtained by processing the original sequencing data (17): (1) We set a window of 25 bp, and if the average window mass value was lower than 20, we cut off the back-end base from the window. If the truncated read length was less than 75% of the original read length, the entire sequence was removed. (2) We removed joint contamination reads. (3) We removed reads containing N. (4) We removed low-complexity reads. Samples were differentiated according to the barcode and primers, and the allowable mismatch number between the barcode sequence and sequencing reads was 0 bp.

High-quality clean data were obtained after removing the low-quality sequencing fragments (reads). Barcode and primer sequences were cut off, and the pairwise reads were merged into tags by overlapping the relationships using FLASH software (v1.2.11; http://ccb.jhu.edu/software/FLASH/index.shtml/FLASH-1.2.11.tar.gz) (18). USEARCH software (v7.0.1090; http://www.drive5.com/usearch) was used to cluster the tags into the operational taxonomic units (OTU) with 97% similarity. The OTUs were assigned taxonomically to the GreenGene Database (V201305; http://greengenes.secondgenome.com) (19) as references for species identification based on the Ramer-Douglas-Peucker classifier algorithm (v2.2) (20).

### Bioinformatics and Statistical Analysis

The statistical analysis was performed using R programming language (v 3.1.1; https://www.r-project.org), SPSS 22.0 software (IBM Corp., Armonk, NY, USA), and GraphPad Prism version 6.01 (GraphPad Software, Inc., San Diego, CA, USA). To analyze the differences in bacteria between the sample groups, the false discovery rate (FDR) control was used for the multiple hypothesis test. *P*<0.05 and FDR<0.05 were considered statistically significant.

### Alpha Diversity

We computed alpha diversity using the OTUs identified from the samples to determine the abundance and homogeneity of the microbial communities (Shannon index and Chao-1 index). To test whether the microbiota diversity difference between the patients and HCs was statistically significant, we applied the analysis of similarity (ANOSIM). ANOSIM was also performed to compare the difference of microbial diversity between groups with different therapeutic responses.

### Beta Diversity

To identify the differences in the microbial community compositions among the samples, we calculated the beta diversity based on unweighted UniFrac and the Bray-Curtis dissimilarity measure. Beta diversity was compared using principal coordinate analysis (PCoA) on all samples. ANOSIM was also performed to compare the difference of beta diversity between groups with different therapeutic responses.

### Differential Flora Analysis

To determine the features differentiating communities of fecal microbiota among the study groups (patients and HCs, groups with different therapeutic responses), we applied linear discriminant analysis (LDA) effect size (LEfSe) and adjusted the P value using the Benjamini-Hochberg method. In our study, an underestimated FDR<0.05 and *P*<0.05 were considered statistically significant. We conducted LEfSe with α of 0.05 (Wilcoxon ranksum test) and assessed the significant effect size threshold of 3.6 on LDA as the critical value.

In order to analyze and identify the bacterial species with abundance differentiation in each group with different therapeutic responses, we applied the Mann-Whitney U test and Kruskal-Wallis test. Continuous clinical variables were compared using χ^2^ or Fisher exact test. Survival analysis was performed using the Kaplan-Meier estimate and log-rank test. The receiver operating characteristic (ROC) curves were used to determine the predictive ability of microbial taxa variation in differentiating patients with progression disease (PD) and non-PD. The cutoff values were estimated at various sensitivities and specificities and were determined at the maximized Youden’s index (Sensitivity+Specificity-1). To identify the correlation between microbiota and clinical biomarkers or drugs, Spearman’s test was performed.

## Results

### Characterization of Study Population

The patient cohort included 130 patients with GI cancers. The demographic and clinical characteristics of the patients are shown in **Table 1**. The patients with cancers were aged 29–75 y (median age 63.5 y) and consisted of 93 males and 37 females. The control cohort included 147 HCs aged 22–74 y (median age 55 y), with 84 males and 63 females. The patient cohort showed a male dominance, which was demonstrated in a previous epidemiological study (2). The age distributions were balanced between the GI tumor group and HC group, as well as the different tumor groups (**Figure S1**). There were 40 cases with esophageal squamous cell carcinoma, 46 cases with gastric adenocarcinoma, and 44 cases with colorectal adenocarcinoma in the patient cohort. Among the patients, 51 were identified as having locally advanced tumors, and 79, distant metastases. Forty patients were treated with an oxaliplatin and fluorouracil-based regimen, which was the most common therapeutic regimen in the patient cohort. Six patients underwent immunotherapy. Four patients only received supportive therapy according to their personal wishes. There were 26 cases of grade 3–4 adverse reactions and complications among the 126 patients.

**Table 1:** Demographic and clinical features of patients with 130 patients with gastrointestinal cancer.

Features	GI cancer patients (n = 130)
Age median (range)	63.5 (29–75)
Gender (M/F)	93/37
Types of tumor	
Esophageal cancer	40 (30.8%)
Gastric cancer	46 (35.4%)
Colorectal cancer	44 (33.8%)
Staging of patients	
Locally advanced	51 (39.2%)
Distant metastases	79 (60.8%)
Antitumor regimen	
Oxaliplatin+5-Fluorouracil	69 (53.1%)
Taxane+Platinum	40 (30.8%)
Irinotecan	5 (3.8%)
Immunotherapy	6 (4.6%)
Others	10 (7.6%)
Adverse reaction of therapy	
Grade 1–2	100 (79.4%)
Grade 3–4	26 (20.6%)
ESR and hsCRP elevation	57 (43.8%)
Lymphopenia	36 (27.7%)
Abnormal lymphocytes subgroups (n=113)	
B cells decreased	65 (57.5%)
CD4^+^T cells decreased	15 (13.3%)
CD8^+^T cells decreased	26 (23.0%)
Naive CD4^+^T cells decreased	86 (76.1%)
CD4^+^CD28^+^T/CD4^+^T decreased	21 (18.6%)
CD8^+^CD38^+^T/CD8^+^T decreased	38 (33.6%)
NK cells decreased	22 (19.5%)
NK cells increased	35 (31.0%)

The baseline inflammatory markers ESR and hsCRP were elevated in 57 cases. Lymphopenia occurred in 36 cases. In total, 113 cases in the patient cohort had baseline lymphocyte subgroup data. Some had significantly decreased T, B, natural killer cell (NK), or lymphocyte subgroups (**Table 1**).

A total of 340 fecal samples were sequenced by 16S rRNA testing. The baseline fecal samples were collected from all 130 patients before treatment. The second fecal samples were collected at the time of disease assessment from 61 patients. Third and fourth fecal samples were collected from one patient due to severe adverse reactions related to immunotherapy. A total of 30,502,042 tags, with an average of 89,712 tags per sample, were obtained from 340 samples. After filtering and clustering, we identified a total of 6,534 OTUs at more than 97% similarity level.

### Comparative Analysis of the Gut Microbiota Between Patients With GI Cancer and HCs

**Figure 1:**
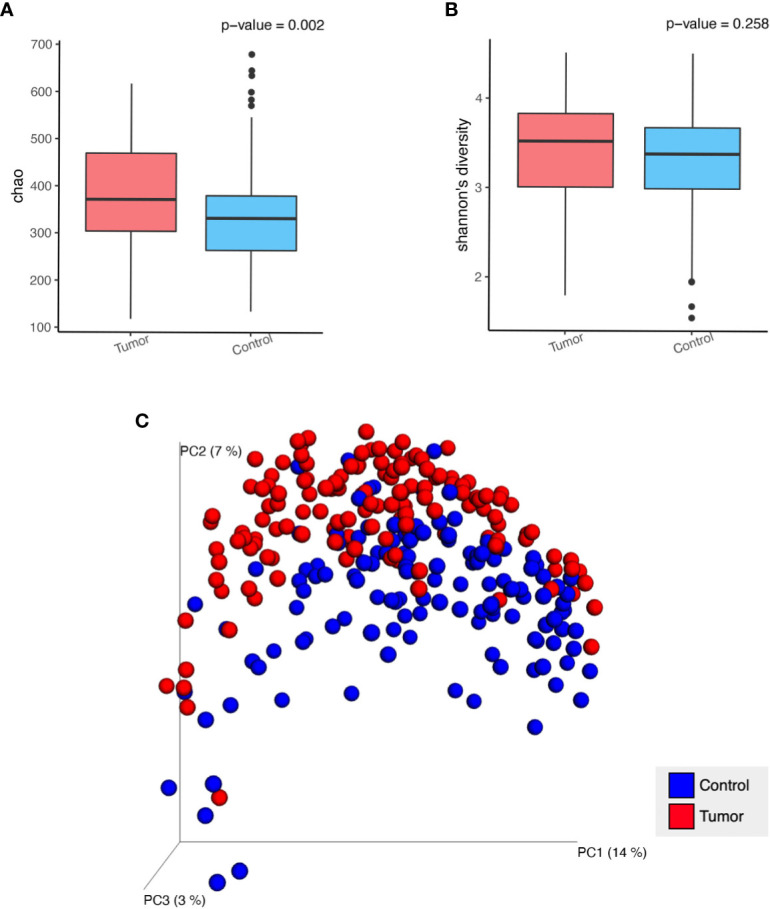
Analysis of alpha diversity and beta diversity in patients with GI cancer (Tumor) compared with healthy controls (Control). **(A)** Chao 1 richness index, **(B)** Shannon index, **(C)** Principal coordinate analysis (PCoA) plot of unweighted Unifrac distances in which the samples are colored by different groups. The percentage of microbial diversity captured by each coordinate is shown.

We compared the alpha diversity of the patient cohort and HCs using the Chao1 and Shannon indices, which reflect richness and diversity, respectively. Boxplots indicated that the Chao1 index (**Figure 1A**) was higher in the patients than in the HCs using the *t*-test (377.0 *vs.* 388.8, *P*=0.00217). There was a similar Shannon index (**Figure 1B**) between the patients and HC cohorts (3.4 *vs.* 3.3, *P*=0.25785). Unifrac-based PCoA of beta diversity evaluated using unweighted analysis showed the significant difference between the HCs and the patients (**Figure 1C**, ANOSIM analysis, R=0.282, *P*=0.001). These data indicated that the richness of fecal microbiota increased, and the composition shifted in the patients.

**Figure 2:**
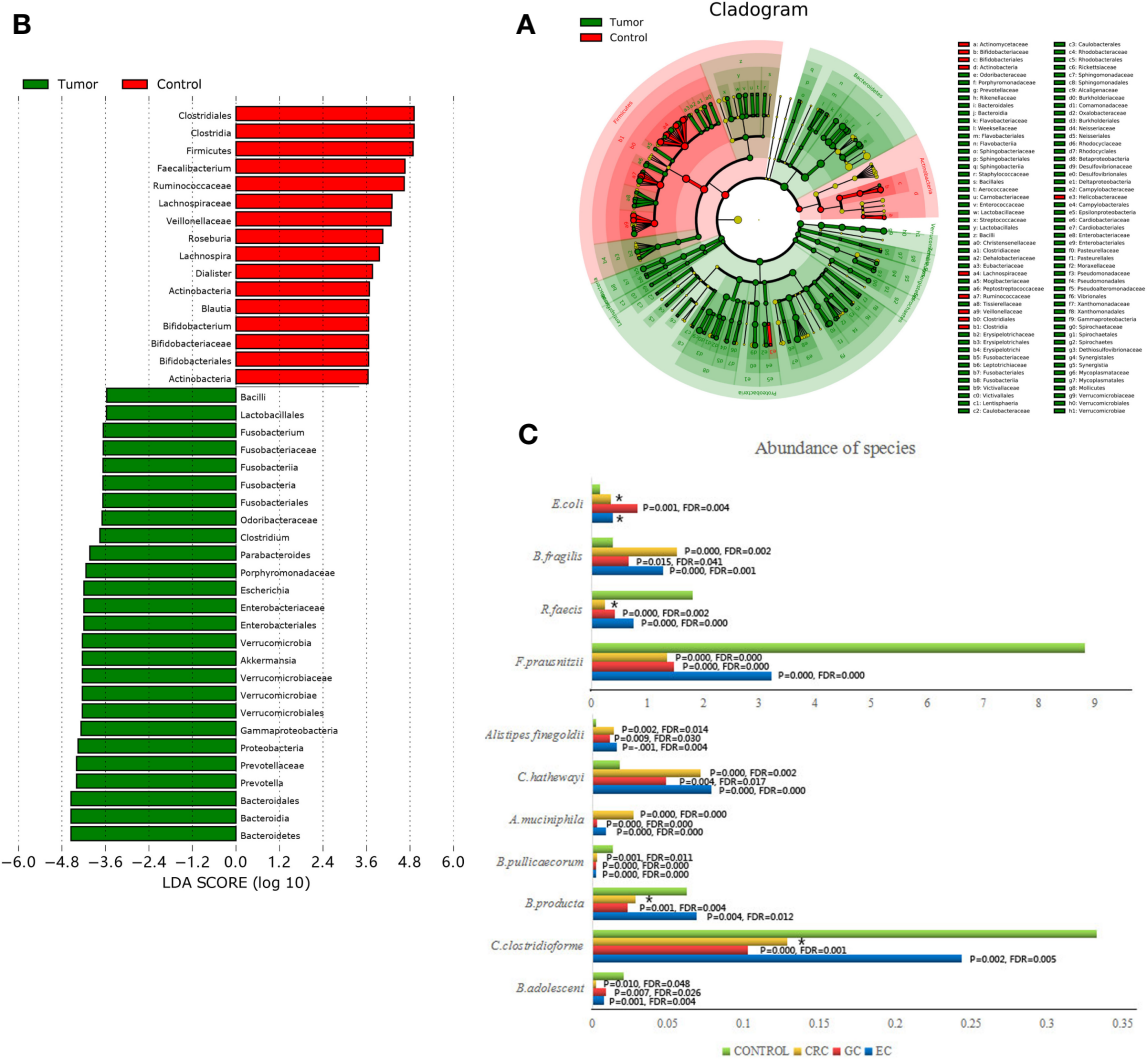
Taxonomic differences were detected between GI cancer patients (Tumor) and healthy controls (Control). **(A)** LEfSe cladogram representing the differentially abundant microbiota genera in comparison. **(B)** Diagram of the LDA scores calculated at species level between GI cancer patients and healthy controls. Only the LDA score >3.6 are shown in the figure. **(C)** Histograms representing the differences between each cancer group (EC, GC, CRC) and healthy control group (Control) separately attributed to each taxon on the species level. * no statistical difference.

We conducted a discriminant analysis using LEfSe to identify the significant differentiating genus of bacteria between study groups (**Figure 2A**). An LDA value of ≥3.6 was used as cutoff value, and the genera with LDA values greater than 3.6 are shown in **Figure 2B**. Compared with the HC group, the relative abundance of *Bacteroides fragilis*, *Escherichia coli*, *Akkermansia muciniphila*, *Clostridium hathewayi*, and *Alistipes finegoldii* were significantly increased in the patient group. On the contrary, *Faecalibacterium prausnitzii*, *Roseburia faecis*, *Clostridium clostridioforme*, *Blautia producta*, *Bifidobacterium adolescent*, and *Butyricicoccus pullicaecorum* taxa were significantly more abundant in the HC group (**Figure 2C**). There was no significant difference in the fecal microbiota among the EC, GC, and CRC groups.

Overall, these results demonstrated that the gut microbial communities differed between the patients and HCs. The major features of the gut microbiota in the patients with EC and GC were similar to those in the patients with CRC.

### Correlations Between Bacterial Abundance Variation and Chemotherapy Efficacy

Chemotherapy efficacy can be assessed in 117 of the patients. We segregated responders (R, n=102) from non-responders (NR, n=15) on the basis of radiographic assessment according to the best clinical response as confirmed by RECIST 1.1. The patients in the R group achieved a favorable response (partial response or stable disease), while the NR group experienced disease progression. The age distribution was balanced between the R and NR groups. We sought to investigate if differences existed between the baseline gut microbiomes of the R and NR groups. To explore this, we compared the diversity of microbiota communities in baseline fecal samples between the R and NR groups. No significant differences were observed in alpha diversity and beta diversity between both groups (**Figure S2**). We next compared the enrichment of OTUs and performed LEfSe to identify differentially abundant taxa in the fecal microbiota of both groups in response to chemotherapy. No major differences were detected in the baseline fecal microbiota between both groups (**Table S1**).

Furthermore, we grouped 117 patients based on their types of tumor (EC: n=33; GC: n=43; and CRC: n=41). In each cancer type cohort, we further divided the patients into R and NR subgroups based on the response to chemotherapy. We analyzed the baseline taxonomic differences between R and NR in the EC, GC, and CRC subgroups, respectively. No significant differences were observed (**Tables S2–S4**).

**Figure 3:**
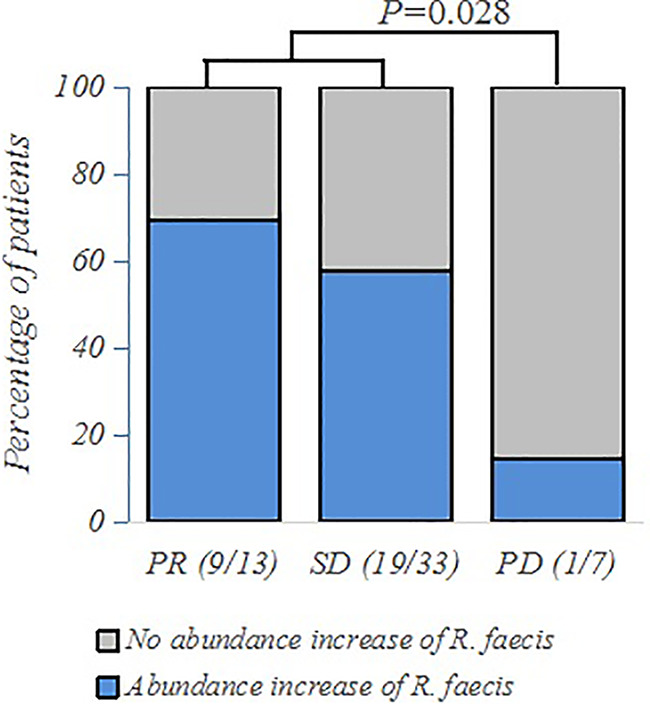
Frequency of patients with abundance increase of *R. faecis* in their feces according to PR (partial response), SD (stable disease), or PD (progressive disease) clinical status, as assessed by 16S rRNA sequencing and analyzed using χ^2^ and Fisher exact tests.

Out of the 117 patients with clinical efficacy data, 53 had fecal samples at two time points: baseline sample (named “baseline”) before chemotherapy started and the second sample at the time of assessment of the therapeutic response (named “treatment”). We hypothesized that the bacterial abundance variation might be associated with the specific therapeutic response to chemotherapy. We observed a trend of abundance increase of *R. faecis* in the R group, whereas there was a decreasing abundance in the NR group. More precisely, the abundance increase of *R. faecis* was observed in 69.2% (9/13) and 57.6% (19/33) of patients exhibiting a partial response (PR) or stable disease (SD), respectively (RECIST 1.1). The increase could only be observed in 14.3% (1/7) patients who were confirmed to have disease progression (PD/NR), which was significantly different from R (PR+SD) patients (*P*=0.028, **Figure 3**). No significant differences of abundance variation of other species between the different efficacy (R or NR) group were demonstrated in our analyses.

To further explore these findings quantitatively, we then defined the increase of relative abundance of bacterial taxa after treatment as a positive value and a decrease as a negative value and calculated the absolute value of relative abundance change of each taxa. We performed the Mann-Whitney U test to observe the differences of abundance variation between groups (R and NR) with different treatment outcomes after chemotherapy. The results demonstrated that the abundance of *R. faecis* in the NR group was more likely to decrease significantly after chemotherapy, while the abundance mostly increased in the R group (*P*=0.024, **Table 2**). No similar trend of abundance variation was observed with other species (**Table 2**).

**Table 2:** Mann-Whitney U test of abundance variation between responder and non-responder groups.

Efficacy	P value						
	*Akkermansiamuciniphila*	*Bacteroides fragilis*	*Bacteroides plebeius*	*Clostridium hathewayi*	*Escherichia coli*	*Faecalibacterium prausnitzii*	*Roseburia faecis*
PR *vs* SD *vs* PD	0.360	0.421	0.219	*0.051*	0.739	0.724	0.073
R *vs* NR	0.888	0.259	0.447	0.148	0.969	0.163	0.024^*^

*Statistically significant.

In an attempt to detect whether the abundance variation of efficacy-related bacteria varies with different types of GI cancers, we grouped 53 patients who had both baseline and treatment samples according to tumor types (EC: n=16; GC: n=21; and CRC: n=16). We also grouped the patients in each cancer type group into PR, SD, and PD subgroups. We analyzed the microbial abundance variation between subgroups with different chemotherapeutic responses in each cancer group. By means of the Kruskal-Wallis test, we determined that the abundance of *R. faecis* in patients with PD in the EC group decreased significantly after chemotherapy, while that in patients with PR increased significantly (*P*=0.019, **Table 3**). Compared with SD, the abundance of *R. faecis* in patients with PR showed an increasing trend (*P*=0.054, **Table 3**) in the EC group. After combining the PR and SD subgroups into one R subgroup, we reanalyzed the abundance variation and found that the abundance variation differences between R and NR were not significant (*P*=0.067, **Table 3**). There was no distinct significant change in abundance of *R. faecis* between the subgroups with different efficacies in the GC and CRC cohorts.

**Table 3:** Kruskal-Wallis test of abundance variation in different gastrointestinal cancer groups according to treatment response.

Tumor type	P value			
	PR *vs* SD *vs* PD		R *vs* NR	
EC	0.010^*^		0.067	
GC	0.232		0.182	
CRC	0.143		0.104	
	P value			
ESO	PR *vs* PD	PR *vs* SD		SD *vs*PD
	0.019^*^	0.054		0.646

*Statistically significant.

### ROC Analysis

**Figure 4:**
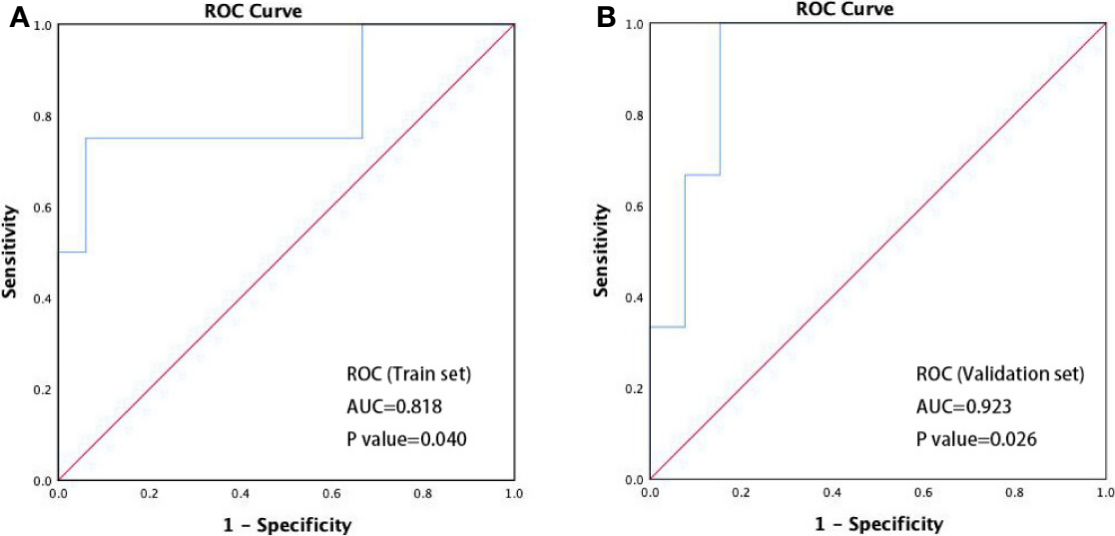
ROC curve discriminating disease progression (PD) *vs.* non-progression (non-PD). **(A)** Performance of prediction models in classifying PD and non-PD in train set (n=37, AUC=0.818, 95% confidence interval 0.536–1.000, P=0.040); **(B)** performance of prediction models in classifying PD and non-PD in validation set (n=16, AUC=0.923, 95% confidence interval 0.786–1.000, P=0.026).

An ROC curve analysis was performed for the abundance changes of *R. faecis* to identify the patients with different responses (PD/non-PD). We defined the increase of relative abundance of bacterial taxa after treatment as a positive value and a decrease as a negative value and calculated the absolute value of relative abundance change of *R. faecis*. We built a classification model on the basis of the features of abundance variation. The model performance was evaluated with an area under the curve (AUC) of ROC with a cross-validation (70% train set, 30% validation set). As shown in **Figure 4A**, the optimal abundance variation of *R. faecis* may be a predictor for distinguishing patients with PD from those with non-PD among the entire patient cohort, with a sensitivity of 75.0% and a specificity of 93.9%. The ROC curve analysis revealed that there was a significantly worse response in patients with abundance reduction >−0.93% from the baseline abundance of *R. faecis* (AUC=0.818, *P*=0.040). The prediction performance was verified in the validation set (**Figure 4B**). The analysis results suggested that the abundance decrease of *R. faecis* after chemotherapy may indicate a worse treatment response. This abundance variation of *R. faecis* was not significantly associated with clinical outcomes in the EC, GC, and CRC groups in a separate analysis (**Figure S3**).

### Prognostic Significance of the Changes in Abundance for Progression-Free Survival

**Figure 5:**
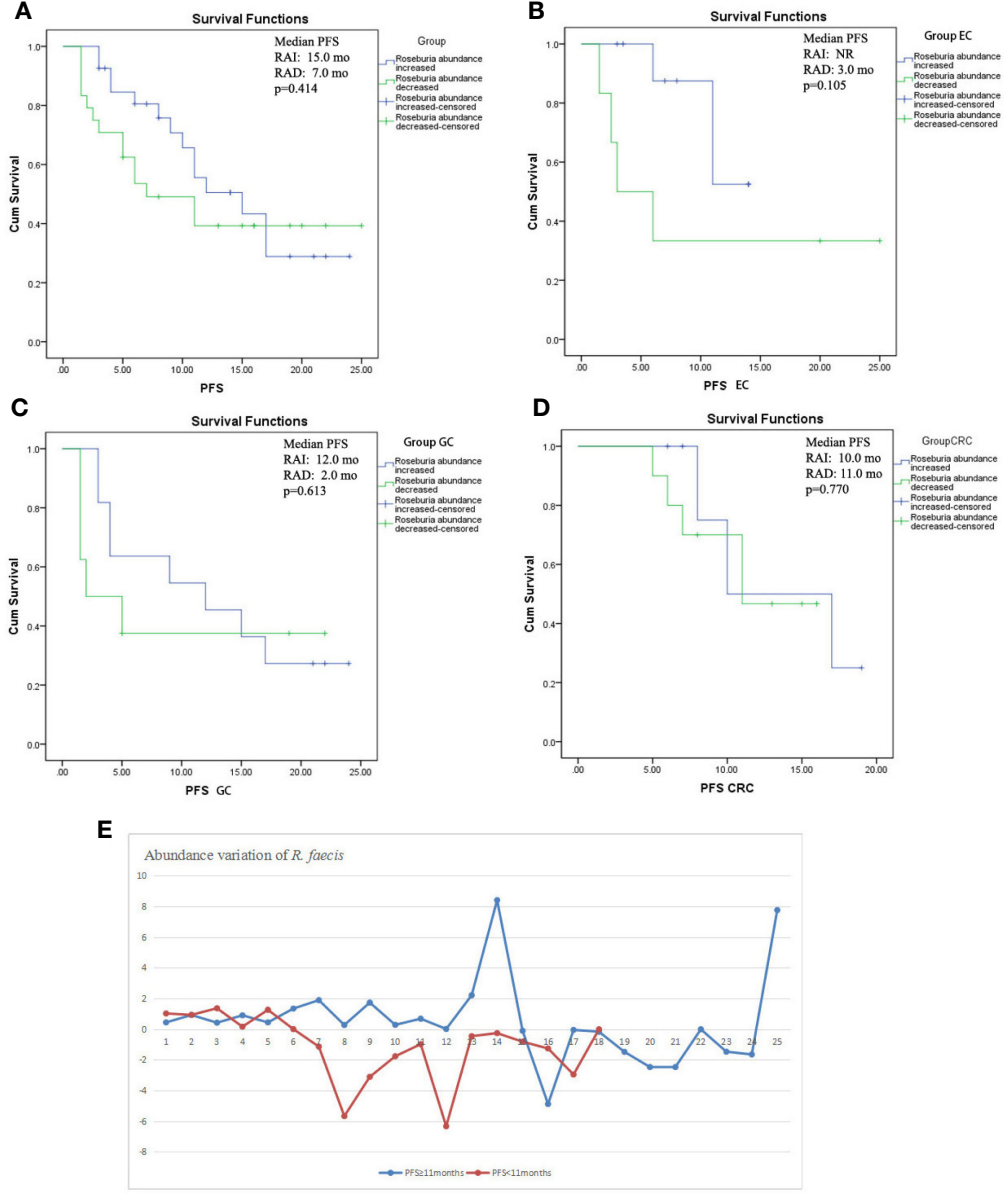
Kaplan-Meier estimates of progression-free survival (PFS) between groups with *R. faecis* abundance increase and abundance decrease. **(A)** PFS in all patients with GI cancer, **(B)** PFS in esophageal cancer patients (EC), **(C)** PFS in gastric cancer patients (GC), **(D)** PFS in colorectal cancer patients (CRC); **(E)** abundance variation of *R. faecis* in patients with PFS ≥11 mo and patients with PFS <11 mo.

From the previous analysis, it could be deduced that the changes of abundance of *R. faecis* may have a predictive value for the chemotherapy response. We compared PFS following chemotherapy to explore whether this finding is significant. All of the patients with an abundance elevation of *R. faecis* after chemotherapy did not have a significantly prolonged PFS *versus* those with an abundance decrease (**Figure 5A**). Of note, the survival curves remained separated from 2 mo to approximately 17 mo before overlapping throughout the follow-up period. Similarly, Kaplan-Meier estimates for the PFS of patients with EC, GC, and CRC were not significantly associated with the *R. faecis* abundance change in subgroup analysis, although the survival curve separated partially in the EC and GC groups (**Figures 5B–D**). A majority of the patients with PFS longer than 11 mo tended to manifest an increase of *R. faecis* after chemotherapy compared to those with PFS <11 months (*P*=0.058, **Figure 5E**).

### Correlations Between *Roseburia* Abundance and Clinical Biomarkers

In an attempt to identify the correlation between microbiota with clinical biomarkers, we analyzed the baseline abundance and abundance variation of *R. faecis* according to different clinical situations, such as TNM stages, sex, age, differentiation, hsCRP, total lymphocytes, and lymphocyte subgroups. Results indicated that the baseline abundance and the variation after chemotherapy were not correlated with these clinical biomarkers. Spearman’s correlation analysis showed a positive correlation between the baseline abundance of *R. faecis* and NK cell proportion (r=0.329, *P*=0.029).

To investigate the link between chemotherapeutic drugs and gut microbiota variation, we examined the microbiota change (6–8 wk after chemotherapy *versus* the baseline) according to different chemo-regimens. There were some differences in abundance variation of some taxa between regimens; however, they were not significant because of the small samples (**Figure S4**).

## Discussion

In recent decades, increasing evidence has shown that gut microbiota plays an important role in health and disease (4), especially in cancer (9). Various studies have shown that gut microbiota are connected with the development of GI cancers (6–8).

We performed data quality control in sequencing processing and age distribution analysis to rule out possible influential factors. There are significant differences in the microbiota in elderly people compared to young adults, as shown by previous studies (20). However, almost all previous studies related to the gut microbiota have been performed on patients classified into segmented age groups based on varying standards. Our results showed that the distribution trend of age was balanced in different groups. Moreover, most of our included patients’ ages ranged from 50 to 80 years; this range was defined as the same age group in most articles (15). We deduced that the differences in age may have little effect on the results in the inter-group comparison.

Our study revealed differing intestinal microbiota structures in patients with GI cancer compared with those of healthy people. Although it is well-recognized that the significant differentiation of gut microbiota exists between patients with CRC and healthy people, it is of great significance to demonstrate the similar microbial features in EC and GC. The abundance of *B. fragilis*, *E. coli*, *A. muciniphila*, *C. hathewayi*, and *Alistipes finegoldii* was found to be higher in patients with GI cancer than in healthy individuals. However, the opposite relationship was observed in the abundance of *F. prausnitzii*, *R. faecis*, *C. clostridioforme*, *B. producta*, *B. adolescent*, and *B. pullicaecorum.* Previous research has reported that the dominant microbiota *F. prausnitzii* can produce butyrate. Butyrate was verified to prevent carcinogenesis by blocking the nuclear factor kappa beta signaling pathway and inducing the activation of T cells. This study also demonstrated the reduction in the abundance of *F. prausnitzii* in the feces of the patients with CRC compared with the healthy participants (21), which is consistent with our study. *C. clostridioforme* activates the intracellular signaling pathways in the inflammatory response (22) and has been reported to decrease in the feces of patients with CRC (23). Melanoma-bearing mice with a high abundance of *Bifidobacterium* in the gut demonstrated a better response to immunotherapy. Therefore, *Bifidobacterium* was considered a protective taxa (11). Similarly, the reduction in gut *Ruminococcus* and *Roseburia* of compared with healthy people was reported (24, 25). *Roseburia* can ferment dietary fiber into butyrate, which was considered to be a protector of the gut (6). These findings suggest that these microbial characteristics in patients with EC, GC, and/or CRC might be useful in identifying novel diagnostic biomarkers in further investigation.

Despite the strong indications for the role of the gut microbiota in carcinogenesis, evidence concerning its role in the context of cancer treatment is currently limited. At the time of writing, the majority of the results concerning interactions between the gut microbiota and cancer therapy originate from studies in the immunotherapy field. As such, clinical research concerning the association between gut microbiota and chemotherapy is also limited. We investigated the role of the fecal microbiota found in the GI cancer cohort in chemotherapy to identify microbiota with an impact on chemotherapy efficacy. To our knowledge, the study published mainly focused on the association between baseline gut microbiota and antitumor response. Results from *in-vitro* studies indicated that a higher abundance of baseline *Lactobacillus plantarum* potentiates the therapeutic response of 5-fluorouracil in chemo-resistant cells (26). Several published articles described the association of baseline gut microbiota with immunotherapy efficacy. Studies have shown that an enriched abundance of *B.* longum (27), *Faecalibacterium* (10, 28, 29), and *A.* muciniphila (30) and a reduced number of *Bacteroides* (28) are positively correlated with favorable immunotherapy outcomes. A study (31) evaluated the fecal microbiome in a cancer patients cohort with multiple cancer types. Gut metagenomic analysis was performed for 26 patients, 4 of whom had CRC. The results demonstrated that *Bacteroides ovatus* and *B. xylanisolvens* were positively correlated with treatment (cytotoxic or targeted therapy with immunotherapy) outcomes. Oral gavage of the responder taxa significantly improved the patient response to erlotinib and induced the expression of CXCL9 and interferon-γ in a murine lung cancer model. This study demonstrated the predictive potential of gut microbiota in the treatment efficacy in various cancer types, including GI cancers, though the sample size was small and the therapeutic modality was inconsistent. Based on the aforementioned studies, we evaluated the baseline abundance of fecal microbiota of R and NR patients. It was revealed that the clinical chemotherapy response was not significantly associated with baseline microbiota. Moreover, no bacterial differences between R and NR were observed in the patients with EC, GC, and CRC.

Apparently, chemotherapies affect the gut microbiota composition and diversity. Therefore, the dysbiosis of the intestinal microbiota might have some consequences for the treatment response. Several studies have reported chemotherapy-induced changes of gut microbiota by collecting fecal samples before and after chemotherapy (32–35). Some species were reported to be affected. *Blautia*, *Roseburia*, *Bifidobacterium*, and *Clostridium clusters IV* and *XIVa* were observed to be decreased during chemotherapy. The variation trend of *Lactobacillus*, *Bacteroides*, *E. coli*, and *F. prausnitzii* were not consistently represented in different studies. On a theoretical basis, we assumed that the variation of microbiota abundance or shift of the microbiota composition during chemotherapy may be related to efficacy. We further investigated the relative abundance variation among subgroups with different therapeutic responses.

Our results showed that the abundance of *R. faecis* was more likely to decline after chemotherapy in the NR esophageal cancer group, whereas the abundance tended to increase significantly in patients with better responses. Detecting cancer progression is crucial in clinical practice in order to adjust the treatment regimen as needed. Using ROC analysis, we evaluated the predictive ability of abundance alteration of *R. faecis* in the prediction of therapeutic response after 6–8 wk of chemotherapy. The analysis revealed significantly worse responses in patients with an abundance reduction >21.2% from the baseline of *R. faecis.* The sensitivity and specificity were 85.7 and 76.1%, respectively, which are acceptable. The application of *R. faecis* for monitoring disease progression might be feasible. As an indicator, the fecal microbiota test is easy to perform and is non-invasive. The PFS superiority was observed before 15 mo, especially in the EC group. We deduced that patients with increased *R. faecis* abundance before 15 mo were less likely to get PD and that the overlap of curves might be related to the change of microbiome diversity after long-term treatment. To the best of our knowledge, the correlation between the change of microbial abundance and the efficacy was rarely investigated in previous studies. It is difficult to distinguish or determine the causal relationship between the bacterial abundance variation and the effectiveness of chemotherapeutic drugs.

*Roseburia* is a member of the *Clostridium coccodis* cluster of the phylum Firmicutes (36). The genus *Roseburia* consists of rod-shaped anaerobic Gram-positive bacteria (37). There are five species in the genus: *R. intestinalis*, *R. hominis*, *R. inulinivorans*, *R. faecis*, and *R. cecicola. Roseburia* spp. are considered to be important parts in the human gut microbiome, with relative abundances of 3.59% in healthy people and 1.56% in patients with CRC (25). *Roseburia* spp. produce SCFAs, especially butyrate, by means of fermenting complex polysaccharides entering the colon (37, 38). *Roseburia* spp. may affect colonic motility and anti-inflammatory and immunity maintenance properties. A previous study found that butyrate producer *Roseburia* spp. may benefit human bodies by preventing inflammatory bowel disease (39), obesity (40), and type II diabetes (41), among other advantages. It has also been suggested that *Roseburia* may be negatively correlated with the development of several tumors, such as CRC (6, 42, 43), cervical cancer (44), nasopharyngeal cancer (45), lung cancer (46), and multiple myeloma (47). Recently, clinical studies of lung cancer showed that patients with lung cancer with a higher abundance of *Roseburia* at the baseline were more likely to have GI reactions to chemotherapy. They also reported that some bacteria with a high abundance at the baseline may related to chemotherapeutic responses, though *Roseburia* was not analyzed (48). We observed the increased abundance of butyrate-producing “beneficial” bacteria *R. faecis* in better-response GI cancer patients, which is compatible with the hypothesis that butyrate may benefit human bodies. The effect of butyrate on the development of tumors may be due to its influence on apoptosis, inhibition of histone deacetylase (49), regulation of vascular endothelial growth factor, and hypoxia inducible factor, so as to inhibit tumor angiogenesis. However, whether the aforementioned mechanisms still play a role in the action of chemotherapeutic drugs needs to be verified by further functional research. Moreover, whether the relatively small changes in gut microbiota have functional consequences, including the influence on butyrate levels, is still a question requiring a metabolomic approach in further research. The exact interaction between chemotherapeutic effect-acting mechanisms and *Roseburia* needs to be clarified. The impact of the chemotherapeutic regimen and clinical factors on the microbiome was not observed in our further evaluation.

Our results demonstrated that the abundance variation of *R. faecis* was significantly associated with the clinical outcome in all of our patients. We did not find significant results in the GC and CRC groups. There are some explanations for this inconsistency. Different GI tumors have different biological behaviors; thus, the mechanisms of the response to chemotherapy may be different. Gut microbiota is complex, which may be influenced by multiple factors. Some previous reports demonstrated that the different gastric microbiota composition was due to the *H. pylori* status (8). The heterogeneity of GC likely also influences the treatment response and may need further investigation. There was only one patient who developed PD in the evaluation of the CRC cohort. The small sample of the PD group may have affected the results of the CRC group. The sample size of the patients available for efficacy analysis was small.

There were some limitations to our study that should be acknowledged. First, the sample size was relatively small, especially the subgroups according to the type of tumor. Furthermore, due to the heterogeneity of the patients’ gut microbiome and the complex impact of food and other factors on microbiota, the alteration of gut microbiota needs to be further analyzed. In addition, our results provide the evidence of association of abundance alteration of *Roseburia* and chemotherapeutic response, but not causality. We did not explore the exact acting mechanism of *Roseburia*. We did not further measure the metabolic products, such as SCFAs, especially butyrate, which might be related to the effect of *Roseburia.* Finally, we did not perform animal research to validate our clinical findings. Thus, we plan to conduct a metabonomical study on patients with GI cancer and an animal study to explore the mechanisms of bacteria in depth in the future.

## Conclusions

The gut microbiome of patients with GI cancer differs from those of healthy people. Moreover, the microbial features in patients with EC, GC, and CRC might be similar, which might be useful in identifying novel diagnostic biomarkers in further investigation.

Most importantly, our study showed features of abundance alteration of *Roseburia* in patients with GI cancer with different chemotherapy efficacies. The results remind us to consider whether *Roseburia* spp. could serve as biomarkers for chemotherapeutic efficacy evaluation. These data also provide us with a theoretical basis for individual therapies for GI cancer. Based on the results, fecal microbiota transplantation might be reconsidered as a promising strategy to enhance the efficacy of cancer chemotherapy.

## Data Availability Statement

The datasets presented in this study can be found in online repositories. The names of the repository/repositories and accession number(s) can be found below: NCBI with BioProject Number 766426 (http://www.ncbi.nlm.nih.gov/bioproject/766426).

## Ethics Statement

The studies involving human participants were reviewed and approved by the ethics committee of Peking Union Medical Hospital. The patients/participants provided their written informed consent to participate in this study.

## Author Contributions

NL designed the study, analyzed the data, and wrote the manuscript. CB, ZS and LZ contributed to the data interpretation and the revision of the manuscript. NL, YG, and XL performed the sample collection. All authors contributed to the article and approved the submitted version.

## Funding

This work was supported by grants from the National Natural Science Foundation of China (No. 61435001) and CAMS Innovation Fund for Medical Sciences (No. 2017-I2M-4-003, No. 2016-I2M-1-001).

## Conflict of Interest

The authors declare that the research was conducted in the absence of any commercial or financial relationships that could be construed as a potential conflict of interest.

## Publisher’s Note

All claims expressed in this article are solely those of the authors and do not necessarily represent those of their affiliated organizations, or those of the publisher, the editors and the reviewers. Any product that may be evaluated in this article, or claim that may be made by its manufacturer, is not guaranteed or endorsed by the publisher.

## Supplementary Material

Supplementary Figure 1 Age distribution in different cancer cohorts. GI, gastrointestinal cancer; ESO, esophageal cancer; GAS, gastric cancer; CRC, colorectal cancer; CONTROL, healthy controls.

Supplementary Figure 2 Comparison of microbial alpha diversity and beta diversity in responder (R) and non-responder (NR) patients. **(A)** Chao 1 richness index, **(B)** Boxplots of Unweighted Unifrac distances of OTUs.

Supplementary Figure 3 ROC curve discriminating disease progression (PD) *vs.* non-progression (non-PD) in EC, GC, and CRC groups in separate analysis.

Supplementary Figure 4 Scatter plots showed abundance variation of *R. faecis* in patients receiving **(A)** TP, **(B)** SOX, **(C)** XELOX, **(D)** FOLFOX, or **(E)** oxaliplatin-containing regimens. TP, paclitaxel, cisplatin; XELOX, oxaliplatin, capetabine; FOLFOX, oxaliplatin, 5-fluorouracil, Leucovorin.

## Abbreviations

GI, gastrointestinal; EC, esophageal cancer; GC, gastric cancer; CRC, colorectal cancer; SCFA, short-chain fatty acids; rRNA, ribosomal RNA; PD-1, anti-programmed cell death receptor-1; PD-L1, anti-programmed cell death ligand-1; PUMCH, Peking Union Medical College Hospital; HC, healthy controls; AJCC, American Joint Committee on Cancer; RECIST, response evaluation criteria in solid tumors; hsCRP, hypersensitive C-reactive protein; ESR, erythrocyte sedimentation rate; OTU, operational taxonomic units; FDR, false discovery rate; ANOSIM, analysis of similarity; PCoA, principal coordinate analysis; LDA, linear discriminant analysis; LEfSe, linear discriminant analysis effect size; ROC, receiver-operating characteristics; PD, progression disease; NK, natural killer cell; R, response; NR, no response; PR, partial response; SD, stable disease; PFS, progression-free survival; NF-kB, nuclear factor kappa beta; 5-FU, 5-fluorouracil; VEGF, vascular endothelial growth factor; FMT, fecal microbiota transplantation.
