# Supplementary figures and images for: The Relationship Between Gut Microbiome Features and Chemotherapy Response in Gastrointestinal Cancer
Source: Front Oncol. 2021 Dec 23;11:781697. doi: 10.3389/fonc.2021.781697 (PMC8733568; doi:10.3389/fonc.2021.781697)

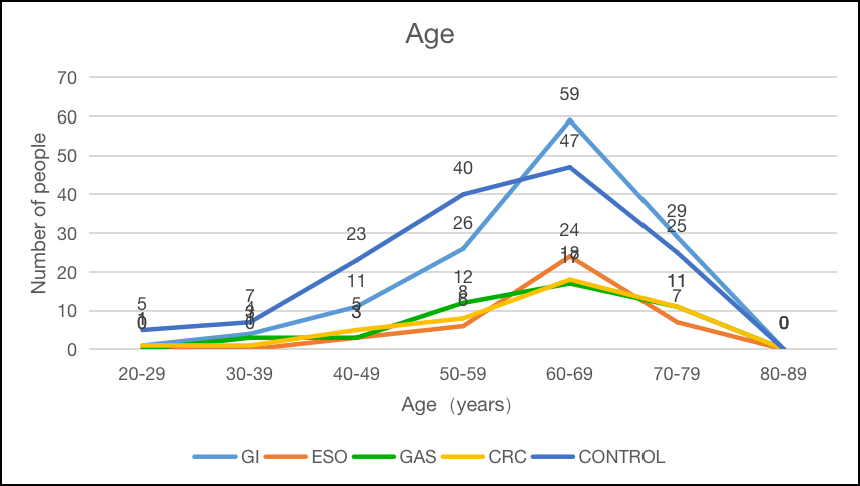

Supplement: Supplementary Figure 1 — Age distribution in different cancer cohorts. GI, gastrointestinal cancer; ESO, esophageal cancer; GAS, gastric cancer; CRC, colorectal cancer; CONTROL, healthy controls. [file Image_1.jpeg]

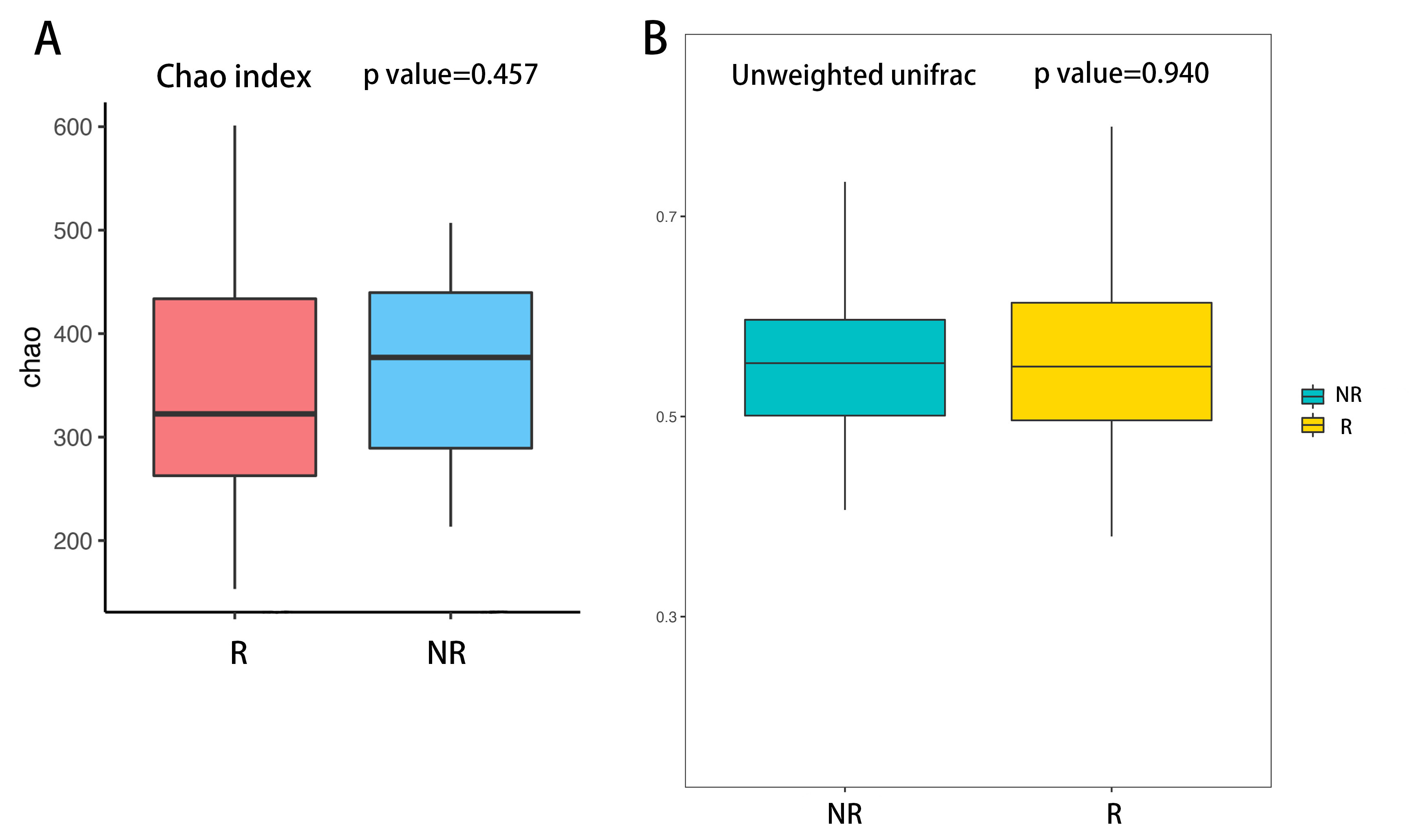

Supplement: Supplementary Figure 2 — Comparison of microbial alpha diversity and beta diversity in responder (R) and non-responder (NR) patients. (A) Chao 1 richness index, (B) Boxplots of Unweighted Unifrac distances of OTUs. [file Image_2.jpeg]

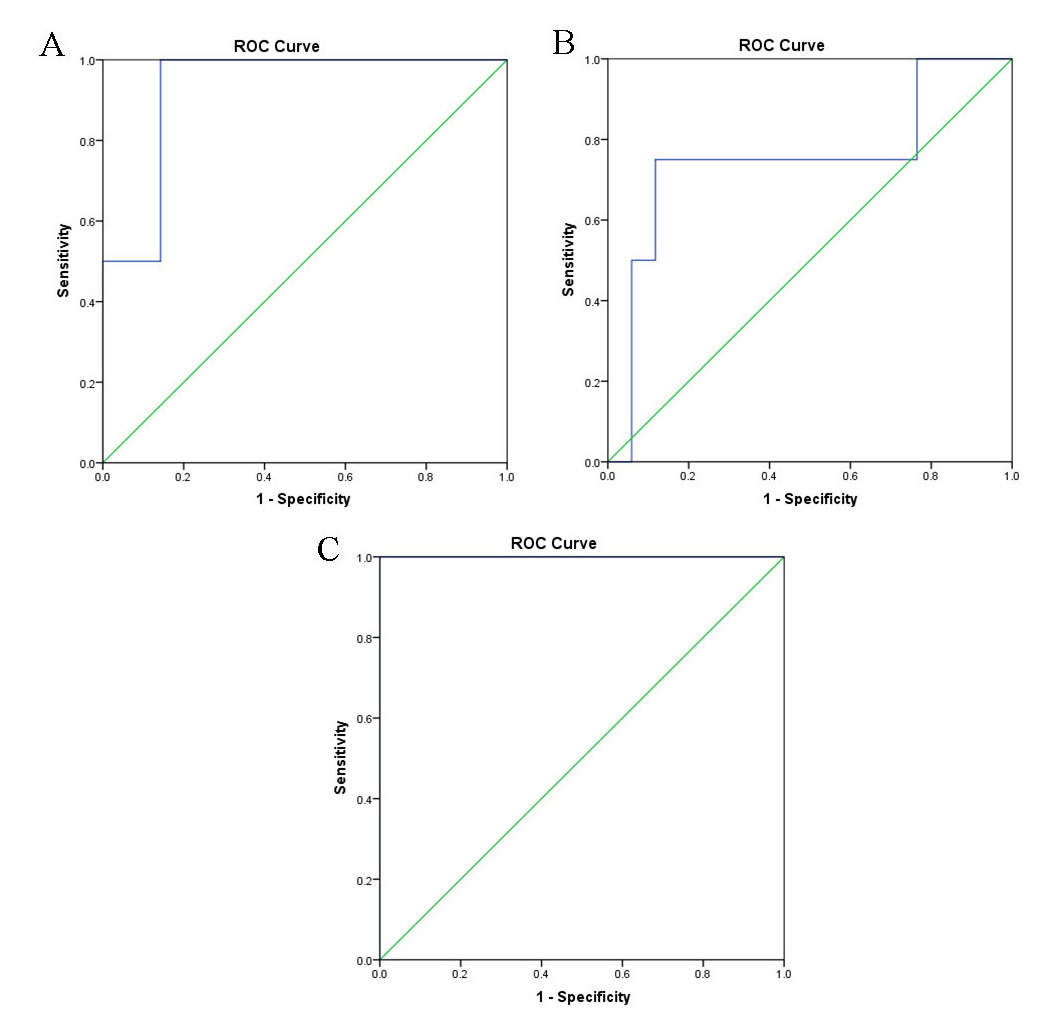

Supplement: Supplementary Figure 3 — ROC curve discriminating disease progression (PD) vs. non-progression (non-PD) in EC, GC, and CRC groups in separate analysis. [file Image_3.jpeg]

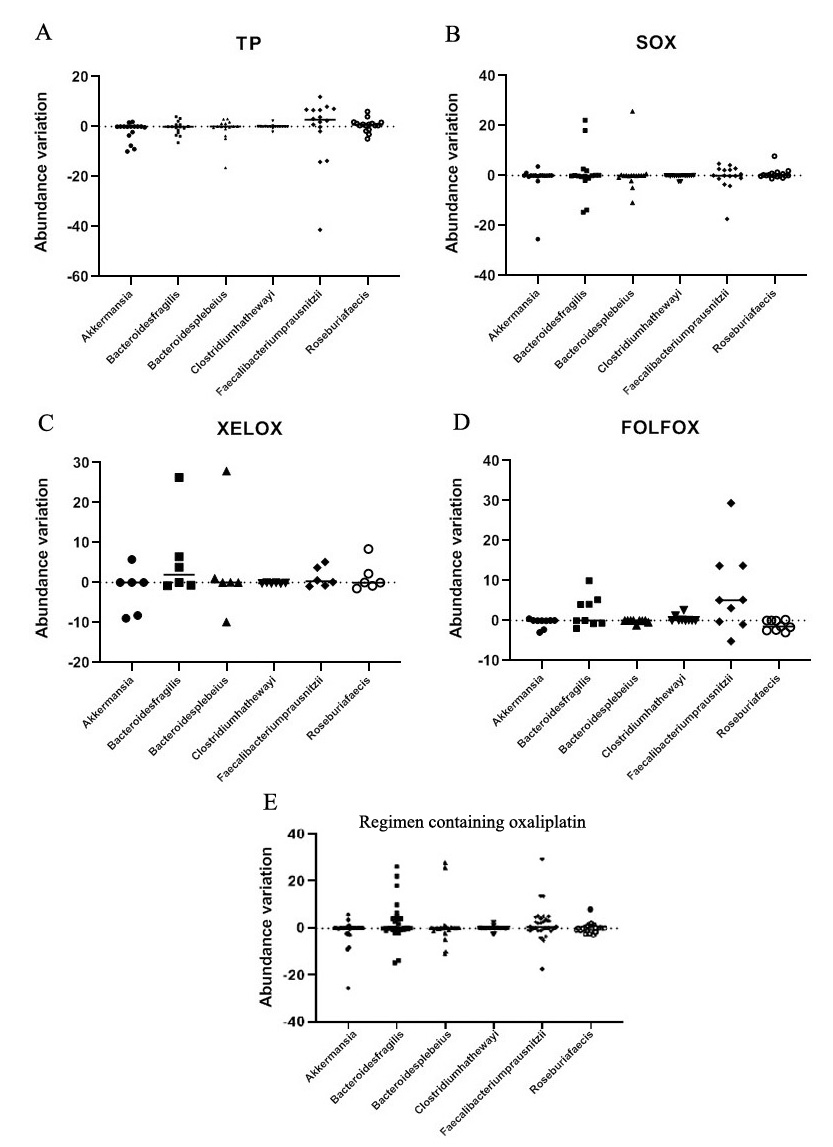

Supplement: Supplementary Figure 4 — Scatter plots showed abundance variation of R. faecis in patients receiving (A) TP, (B) SOX, (C) XELOX, (D) FOLFOX, or (E) oxaliplatin-containing regimens. TP, paclitaxel, cisplatin; XELOX, oxaliplatin, capetabine; FOLFOX, oxaliplatin, 5-fluorouracil, Leucovorin. [file Image_4.jpeg]
