# Supplementary material for: The Relationship Between Gut Microbiome Features and Chemotherapy Response in Gastrointestinal Cancer
Source: Front Oncol. 2021 Dec 23;11:781697. doi: 10.3389/fonc.2021.781697 (PMC8733568; doi:10.3389/fonc.2021.781697)
Supplement: Supplementary file 5 [file Table_1.doc]

**Supplemntal Table 1. Baseline fecal microbiota between R and NR patients with GI cancers (n=117)**

| **Species** | **Median**  **(NR)** | **IQR**  **(NR)** | **Median**  **(R)** | **IQR**  **(R)** | **p.value** | **FDR** |
| --- | --- | --- | --- | --- | --- | --- |
| Acinetobacter_guillouiae | 0 | 0 | 0 | 0 | 0.865448 | 0.97993 |
| Aggregatibacter_segnis | 0 | 0.002827 | 0 | 0.002133 | 0.802777 | 0.97993 |
| Akkermansia_muciniphila | 0.304383 | 0.901551 | 0.010197 | 0.691152 | 0.625418 | 0.943084 |
| Alistipes_finegoldii | 0.012791 | 0.019454 | 0.01325 | 0.042649 | 0.70229 | 0.945658 |
| Alistipes_indistinctus | 0.047943 | 0.141897 | 0.034756 | 0.136169 | 0.381863 | 0.943084 |
| Arcobacter_cryaerophilus | 0 | 0 | 0 | 0 | 0.213582 | 0.943084 |
| Atopobium_vaginae | 0 | 0 | 0 | 0 | 0.694545 | 0.945658 |
| Bacteroides_caccae | 0.184029 | 0.380244 | 0.198311 | 0.82721 | 0.849105 | 0.97993 |
| Bacteroides_coprophilus | 0 | 0 | 0 | 0 | 0.979772 | 0.994395 |
| Bacteroides_eggerthii | 0.152403 | 0.657423 | 0.133064 | 0.473702 | 0.632269 | 0.943084 |
| Bacteroides_fragilis | 1.013223 | 1.636596 | 0.644737 | 2.503636 | 0.786465 | 0.972357 |
| Bacteroides_nordii | 0 | 0 | 0 | 0 | 0.665706 | 0.943084 |
| Bacteroides_ovatus | 1.207424 | 1.404873 | 0.541529 | 2.595593 | 0.849145 | 0.97993 |
| Bacteroides_plebeius | 0.040567 | 3.227937 | 0.007634 | 1.704528 | 0.943007 | 0.97993 |
| Bacteroides_uniformis | 0.428102 | 0.404007 | 0.527745 | 2.780439 | 0.903632 | 0.97993 |
| Barnesiella_intestinihominis | 0 | 0 | 0 | 0 | 0.524394 | 0.943084 |
| Bdellovibrio_bacteriovorus | 0 | 0 | 0 | 0 | 0.665706 | 0.943084 |
| Bifidobacterium_adolescentis | 0.00527 | 0.085196 | 0.009806 | 0.055707 | 0.907424 | 0.97993 |
| Blautia_obeum | 0.038109 | 0.060543 | 0.037391 | 0.108555 | 0.529498 | 0.943084 |
| Blautia_producta | 0.017036 | 0.115781 | 0.015222 | 0.067933 | 0.62938 | 0.943084 |
| Brachybacterium  _conglomeratum | 0 | 0 | 0 | 0 | 0.524394 | 0.943084 |
| Brevundimonas_vesicularis | 0 | 0 | 0 | 0 | 0.354052 | 0.943084 |
| Bulleidia_moorei | 0 | 0.000785 | 0 | 0.00194 | 0.387576 | 0.943084 |
| Butyricicoccus_pullicaecorum | 0.058077 | 0.088607 | 0.061557 | 0.134654 | 0.786445 | 0.972357 |
| Butyrivibrio_crossotus | 0 | 0 | 0 | 0 | 0.175749 | 0.943084 |
| Campylobacter_ureolyticus | 0 | 0 | 0 | 0 | 0.148122 | 0.943084 |
| Capnocytophaga_ochracea | 0 | 0 | 0 | 0 | 0.524394 | 0.943084 |
| Cardiobacterium_valvarum | 0 | 0 | 0 | 0 | 1 | 1 |
| Clostridium_aldenense | 0.003837 | 0.013264 | 0.006452 | 0.03743 | 0.304143 | 0.943084 |
| Clostridium_bifermentans | 0 | 0 | 0 | 0 | 0.665706 | 0.943084 |
| Clostridium_butyricum | 0 | 0 | 0 | 0 | 0.524394 | 0.943084 |
| Clostridium_celatum | 0.001255 | 0.004587 | 0.010058 | 0.088236 | 0.068459 | 0.943084 |
| Clostridium_cellulolyticum | 0 | 0 | 0 | 0 | 0.665706 | 0.943084 |
| Clostridium_citroniae | 0.054509 | 0.125629 | 0.02763 | 0.156705 | 0.699284 | 0.945658 |
| Clostridium_clostridioforme | 0.092922 | 0.256183 | 0.183545 | 0.427893 | 0.931083 | 0.97993 |
| Clostridium_colicanis | 0 | 0 | 0 | 0 | 0.459769 | 0.943084 |
| Clostridium_colinum | 0 | 0 | 0 | 0.008623 | 0.34187 | 0.943084 |
| Clostridium_hathewayi | 0.03688 | 0.071642 | 0.041124 | 0.115391 | 0.84003 | 0.97993 |
| Clostridium_hungatei | 0 | 0 | 0 | 0 | 0.657234 | 0.943084 |
| Clostridium_lavalense | 0.004058 | 0.035537 | 0.009141 | 0.038373 | 0.958314 | 0.97993 |
| Clostridium_methylpentosum | 0.002 | 0.004407 | 0.00123 | 0.005309 | 0.746878 | 0.949303 |
| Clostridium_neonatale | 0 | 0 | 0 | 0 | 0.427593 | 0.943084 |
| Clostridium_paraputrificum | 0 | 0 | 0 | 0.002012 | 0.312811 | 0.943084 |
| Clostridium_perfringens | 0 | 0.003151 | 0 | 0.002142 | 0.949926 | 0.97993 |
| Clostridium_ramosum | 0 | 0.048168 | 0.002052 | 0.034111 | 0.532236 | 0.943084 |
| Clostridium_ruminantium | 0 | 0.003015 | 0 | 0.012064 | 0.55361 | 0.943084 |
| Clostridium_sordellii | 0 | 0 | 0 | 0 | 0.640864 | 0.943084 |
| Clostridium_spiroforme | 0 | 0 | 0 | 0.002275 | 0.060258 | 0.943084 |
| Clostridium_symbiosum | 0.000974 | 0.013164 | 0.003966 | 0.036216 | 0.245159 | 0.943084 |
| Collinsella_aerofaciens | 0.034005 | 0.032119 | 0.017931 | 0.050736 | 0.175989 | 0.943084 |
| Collinsella_stercoris | 0 | 0 | 0 | 0 | 0.856093 | 0.97993 |
| Coprococcus_catus | 0.081823 | 0.148742 | 0.035142 | 0.089696 | 0.29853 | 0.943084 |
| Coprococcus_eutactus | 0 | 0.225489 | 0 | 0.04548 | 0.658262 | 0.943084 |
| Corynebacterium_durum | 0 | 0 | 0 | 0 | 0.130216 | 0.943084 |
| Defluviitalea_saccharophila | 0.007856 | 0.015281 | 0.002116 | 0.011303 | 0.401164 | 0.943084 |
| Desulfovibrio_D168 | 0 | 0 | 0 | 0.008334 | 0.277734 | 0.943084 |
| Desulfurispirillum  _alkaliphilum | 0 | 0 | 0 | 0 | 0.665706 | 0.943084 |
| Dorea_formicigenerans | 0.040584 | 0.063659 | 0.030198 | 0.061291 | 0.150499 | 0.943084 |
| Eggerthella_lenta | 0.00188 | 0.003393 | 0 | 0.001821 | 0.061733 | 0.943084 |
| Elizabethkingia  _meningoseptica | 0 | 0 | 0 | 0 | 0.665706 | 0.943084 |
| Enterococcus_casseliflavus | 0 | 0 | 0 | 0 | 0.208624 | 0.943084 |
| Escherichia_coli | 0.050008 | 0.511298 | 0.639161 | 4.596507 | 0.02978 | 0.943084 |
| Eubacterium_biforme | 0 | 0.262908 | 0 | 0.001987 | 0.295961 | 0.943084 |
| Eubacterium_cylindroides | 0 | 0 | 0 | 0 | 0.24466 | 0.943084 |
| Eubacterium_dolichum | 0.003142 | 0.010345 | 0.004032 | 0.040605 | 0.569853 | 0.943084 |
| Faecalibacterium_prausnitzii | 2.122598 | 4.197264 | 1.683159 | 3.685987 | 0.198668 | 0.943084 |
| Flavobacterium_gelidilacus | 0 | 0 | 0 | 0 | 0.921188 | 0.97993 |
| Flavobacterium_succinicans | 0 | 0 | 0 | 0 | 0.427593 | 0.943084 |
| Gemmiger_formicilis | 0.181009 | 0.563261 | 0.048892 | 0.223567 | 0.098547 | 0.943084 |
| Haemophilus_parainfluenzae | 0.042421 | 0.239332 | 0.02111 | 0.150163 | 0.334686 | 0.943084 |
| Kingella_potus | 0 | 0 | 0 | 0 | 0.247996 | 0.943084 |
| Kocuria_palustris | 0 | 0 | 0 | 0 | 0.741503 | 0.949303 |
| Lachnoanaerobaculum_orale | 0 | 0.000628 | 0 | 0.001354 | 0.65843 | 0.943084 |
| Lactobacillus_delbrueckii | 0 | 0 | 0 | 0 | 0.01236 | 0.84048 |
| Lactobacillus_helveticus | 0 | 0.003393 | 0 | 0 | 0.010213 | 0.84048 |
| Lactobacillus_iners | 0 | 0 | 0 | 0 | 0.524394 | 0.943084 |
| Lactobacillus_mucosae | 0 | 0.005268 | 0 | 0 | 0.02656 | 0.943084 |
| Lactobacillus_reuteri | 0 | 0.026247 | 0 | 0 | 0.045854 | 0.943084 |
| Lactobacillus_salivarius | 0.004259 | 0.115348 | 0 | 0.007703 | 0.104842 | 0.943084 |
| Lactobacillus_zeae | 0 | 0 | 0 | 0 | 0.236155 | 0.943084 |
| Lactococcus_garvieae | 0 | 0 | 0 | 0 | 0.741277 | 0.949303 |
| Malus_x_domestica | 0 | 0 | 0 | 0 | 0.741503 | 0.949303 |
| Massilia_haematophila | 0 | 0 | 0 | 0 | 0.665706 | 0.943084 |
| Morganella_morganii | 0 | 0 | 0 | 0 | 0.427593 | 0.943084 |
| Moryella_indoligenes | 0 | 0 | 0 | 0 | 0.427593 | 0.943084 |
| Neisseria_subflava | 0 | 0.000628 | 0.001672 | 0.004056 | 0.103702 | 0.943084 |
| Olsenella_umbonata | 0 | 0 | 0 | 0 | 0.694545 | 0.945658 |
| Oryza_sativa_Indica_Group | 0 | 0 | 0 | 0 | 0.175749 | 0.943084 |
| Oscillospira_guilliermondii | 0 | 0.008059 | 0 | 0 | 0.223081 | 0.943084 |
| Oxalobacter_formigenes | 0.00188 | 0.038249 | 0.003652 | 0.023803 | 0.957106 | 0.97993 |
| Papillibacter_cinnamivorans | 0 | 0 | 0 | 0 | 0.389923 | 0.943084 |
| Parabacteroides_distasonis | 0.682477 | 0.919475 | 0.590392 | 1.550636 | 0.885412 | 0.97993 |
| Parabacteroides_gordonii | 0 | 0 | 0 | 0 | 0.36579 | 0.943084 |
| Paracoccus_aminovorans | 0 | 0 | 0 | 0 | 0.989644 | 0.996975 |
| Paracoccus_marcusii | 0 | 0 | 0 | 0 | 0.943661 | 0.97993 |
| Paraeggerthella  _hongkongensis | 0 | 0 | 0 | 0 | 0.459769 | 0.943084 |
| Porphyromonas_endodontalis | 0 | 0 | 0 | 0 | 0.175749 | 0.943084 |
| Prevotella_copri | 0.001425 | 28.950639 | 0.003983 | 0.147981 | 0.701599 | 0.945658 |
| Prevotella_intermedia | 0 | 0 | 0 | 0 | 0.08837 | 0.943084 |
| Prevotella_melaninogenica | 0 | 0 | 0 | 0 | 0.381241 | 0.943084 |
| Prevotella_nanceiensis | 0 | 0 | 0 | 0 | 0.524394 | 0.943084 |
| Prevotella_nigrescens | 0 | 0 | 0 | 0 | 0.524394 | 0.943084 |
| Prevotella_stercorea | 0 | 0.340792 | 0 | 0.00139 | 0.25804 | 0.943084 |
| Prevotella_tannerae | 0 | 0 | 0 | 0 | 0.78207 | 0.972357 |
| Propionibacterium_acnes | 0 | 0 | 0 | 0 | 0.427593 | 0.943084 |
| Pseudomonas_stutzeri | 0 | 0 | 0 | 0 | 0.818318 | 0.97993 |
| Psychrobacter_pulmonis | 0 | 0 | 0 | 0 | 0.044228 | 0.943084 |
| Pyramidobacter_piscolens | 0 | 0.005641 | 0 | 0.005344 | 0.950508 | 0.97993 |
| Robinsoniella_peoriensis | 0 | 0 | 0 | 0 | 0.948253 | 0.97993 |
| Roseburia_faecis | 1.883776 | 2.11702 | 0.644948 | 2.662349 | 0.270856 | 0.943084 |
| Roseburia_inulinivorans | 0 | 0 | 0 | 0 | 0.657234 | 0.943084 |
| Rothia_dentocariosa | 0 | 0 | 0 | 0 | 0.931722 | 0.97993 |
| Rothia_mucilaginosa | 0 | 0.001642 | 0.000715 | 0.002611 | 0.232892 | 0.943084 |
| Ruminococcus_albus | 0 | 0 | 0 | 0 | 0.247996 | 0.943084 |
| Ruminococcus_bromii | 0.21902 | 0.938306 | 0.319995 | 1.523862 | 0.737306 | 0.949303 |
| Ruminococcus_callidus | 0.016003 | 0.068021 | 0.010351 | 0.065804 | 0.261261 | 0.943084 |
| Ruminococcus_flavefaciens | 0 | 0 | 0 | 0 | 0.741503 | 0.949303 |
| Ruminococcus_gnavus | 0.234954 | 0.418942 | 0.196713 | 0.285971 | 0.52983 | 0.943084 |
| Ruminococcus_torques | 0.12987 | 0.354642 | 0.045398 | 0.189467 | 0.2955 | 0.943084 |
| Shinella_granuli | 0 | 0 | 0 | 0 | 0.427593 | 0.943084 |
| Shuttleworthia_satelles | 0 | 0 | 0 | 0 | 0.504703 | 0.943084 |
| Sphingobacterium_mizutaii | 0 | 0 | 0 | 0 | 0.356743 | 0.943084 |
| Staphylococcus_succinus | 0 | 0 | 0 | 0 | 0.893468 | 0.97993 |
| Stenotrophomonas_  acidaminiphila | 0 | 0 | 0 | 0 | 0.099083 | 0.943084 |
| Streptococcus_anginosus | 0.006088 | 0.038921 | 0.002022 | 0.019291 | 0.103259 | 0.943084 |
| Streptococcus_infantis | 0.023357 | 0.047766 | 0.018028 | 0.079943 | 0.522287 | 0.943084 |
| Streptococcus_luteciae | 0 | 0.00227 | 0 | 0.002082 | 0.881915 | 0.97993 |
| Streptococcus_sobrinus | 0 | 0.002945 | 0 | 0 | 0.158285 | 0.943084 |
| Succinatimonas_hippei | 0 | 0 | 0 | 0 | 0.665706 | 0.943084 |
| Treponema_amylovorum | 0 | 0 | 0 | 0 | 0.524394 | 0.943084 |
| Treponema_socranskii | 0 | 0 | 0 | 0 | 0.427593 | 0.943084 |
| Unclassified | 64.185779 | 34.215637 | 57.411281 | 24.719996 | 0.436494 | 0.943084 |
| Veillonella_dispar | 0.150198 | 0.19418 | 0.024216 | 0.223193 | 0.436225 | 0.943084 |
| Veillonella_parvula | 0.008518 | 0.026402 | 0.003876 | 0.027939 | 0.645663 | 0.943084 |
| Vibrio_cholerae | 0 | 0 | 0 | 0 | 0.665706 | 0.943084 |
| Victivallis_vadensis | 0 | 0.002198 | 0 | 0.002067 | 0.949302 | 0.97993 |
