# Supplementary material for: The Relationship Between Gut Microbiome Features and Chemotherapy Response in Gastrointestinal Cancer
Source: Front Oncol. 2021 Dec 23;11:781697. doi: 10.3389/fonc.2021.781697 (PMC8733568; doi:10.3389/fonc.2021.781697)
Supplement: Supplementary file 6 [file Table_2.doc]

**Supplemntal Table 2.** Baseline fecal microbiota between R and NR in esophageal cancer (EC) group (n=33)

| **Species** | **Median**  **(NR.EC)** | **IQR**  **(NR.EC)** | **Median**  **(R.EC)** | **IQR**  **(R.EC)** | **p.value** | **FDR** |
| --- | --- | --- | --- | --- | --- | --- |
| Acinetobacter_guillouiae | 0 | 0 | 0 | 0 | 1 | 1 |
| Aggregatibacter_segnis | 0 | 0.003142 | 0 | 0 | 0.20635 | 0.941582 |
| Akkermansia_muciniphila | 0.458509 | 1.016339 | 0.013131 | 2.307225 | 0.862924 | 0.990733 |
| Alistipes_finegoldii | 0.012281 | 0.021854 | 0.012289 | 0.037948 | 0.918519 | 0.990733 |
| Alistipes_indistinctus | 0.139384 | 0.190475 | 0.027058 | 0.079568 | 0.184403 | 0.941582 |
| Arcobacter_cryaerophilus | 0 | 0 | 0 | 0 | 0.689157 | 0.941582 |
| Atopobium_vaginae | 0 | 0 | 0 | 0 | 0.790241 | 0.974631 |
| Bacteroides_caccae | 0.300093 | 0.207149 | 0.31751 | 1.128793 | 0.767137 | 0.967639 |
| Bacteroides_coprophilus | 0 | 0 | 0 | 0.319068 | 0.637094 | 0.941582 |
| Bacteroides_eggerthii | 0.152403 | 0.687415 | 0.177177 | 0.342234 | 0.918817 | 0.990733 |
| Bacteroides_fragilis | 1.013223 | 1.575987 | 0.448663 | 1.96329 | 0.869415 | 0.990733 |
| Bacteroides_ovatus | 0.762015 | 1.338354 | 0.534888 | 2.339992 | 0.530435 | 0.941582 |
| Bacteroides_plebeius | 0 | 0 | 1.500267 | 5.349147 | 0.045423 | 0.70834 |
| Bacteroides_uniformis | 0.307805 | 0.191601 | 0.400705 | 1.40289 | 0.767137 | 0.967639 |
| Bdellovibrio_bacteriovorus | 0 | 0 | 0 | 0 | 0.689157 | 0.941582 |
| Bifidobacterium_adolescentis | 0.003142 | 0.030704 | 0.007354 | 0.041591 | 0.810035 | 0.978247 |
| Blautia_obeum | 0.083924 | 0.059789 | 0.04098 | 0.129019 | 0.518589 | 0.941582 |
| Blautia_producta | 0.017036 | 0.14769 | 0.038242 | 0.076997 | 0.810799 | 0.978247 |
| Bulleidia_moorei | 0.001571 | 0.001948 | 0 | 0.000264 | 0.092703 | 0.941582 |
| Butyricicoccus_pullicaecorum | 0.058077 | 0.016039 | 0.091642 | 0.127784 | 0.446678 | 0.941582 |
| Butyrivibrio_crossotus | 0 | 0 | 0 | 0 | 0.689157 | 0.941582 |
| Campylobacter_ureolyticus | 0 | 0 | 0 | 0 | 0.516003 | 0.941582 |
| Capnocytophaga_ochracea | 0 | 0 | 0 | 0 | 0.689157 | 0.941582 |
| Cardiobacterium_valvarum | 0 | 0 | 0 | 0 | 0.516003 | 0.941582 |
| Clostridium_aldenense | 0 | 0.012281 | 0.004302 | 0.021113 | 0.39956 | 0.941582 |
| Clostridium_celatum | 0.063884 | 0.156041 | 0.002671 | 0.044146 | 0.559367 | 0.941582 |
| Clostridium_citroniae | 0.014907 | 0.116674 | 0.026158 | 0.111797 | 0.29224 | 0.941582 |
| Clostridium_clostridioforme | 0.092922 | 0.751449 | 0.233911 | 0.468039 | 0.972882 | 0.990733 |
| Clostridium_colinum | 0 | 0.00213 | 0 | 0.016102 | 0.788362 | 0.974631 |
| Clostridium_hathewayi | 0.055523 | 0.532914 | 0.028557 | 0.118786 | 0.945826 | 0.990733 |
| Clostridium_hungatei | 0 | 0 | 0 | 0 | 0.673685 | 0.941582 |
| Clostridium_lavalense | 0.077783 | 0.098759 | 0.00732 | 0.021948 | 0.373906 | 0.941582 |
| Clostridium_methylpentosum | 0.003896 | 0.008188 | 0 | 0.005324 | 0.432245 | 0.941582 |
| Clostridium_neonatale | 0 | 0 | 0 | 0 | 0.689157 | 0.941582 |
| Clostridium_paraputrificum | 0 | 0.006141 | 0 | 0.003835 | 0.936987 | 0.990733 |
| Clostridium_perfringens | 0 | 0 | 0 | 0 | 0.961236 | 0.990733 |
| Clostridium_ramosum | 0 | 0 | 0.002912 | 0.042189 | 0.271066 | 0.941582 |
| Clostridium_ruminantium | 0.004713 | 0.070274 | 0 | 0.001576 | 0.179535 | 0.941582 |
| Clostridium_spiroforme | 0 | 0 | 0 | 0.003626 | 0.188656 | 0.941582 |
| Clostridium_symbiosum | 0.000974 | 0.018422 | 0.001347 | 0.00719 | 0.749323 | 0.967639 |
| Collinsella_aerofaciens | 0.044525 | 0.285525 | 0.010878 | 0.056032 | 0.126078 | 0.941582 |
| Collinsella_stercoris | 0 | 0 | 0 | 0 | 0.057433 | 0.70834 |
| Coprococcus_catus | 0.128956 | 0.075294 | 0.079254 | 0.12935 | 0.563553 | 0.941582 |
| Coprococcus_eutactus | 0 | 0 | 0 | 0.06117 | 0.420639 | 0.941582 |
| Corynebacterium_durum | 0 | 0.001936 | 0 | 0 | 0.030339 | 0.70834 |
| Defluviitalea_saccharophila | 0.016375 | 0.017698 | 0.00102 | 0.011875 | 0.214006 | 0.941582 |
| Desulfovibrio_D168 | 0 | 0 | 0 | 0 | 0.516003 | 0.941582 |
| Dorea_formicigenerans | 0.038718 | 0.015955 | 0.040052 | 0.054559 | 0.865032 | 0.990733 |
| Eggerthella_lenta | 0.000974 | 0.006141 | 0 | 0.002004 | 0.432245 | 0.941582 |
| Enterococcus_casseliflavus | 0 | 0 | 0 | 0 | 0.516003 | 0.941582 |
| Escherichia_coli | 0.04085 | 0.303148 | 0.308132 | 2.689299 | 0.475554 | 0.941582 |
| Eubacterium_biforme | 0 | 0.138263 | 0 | 0.000403 | 0.440126 | 0.941582 |
| Eubacterium_dolichum | 0.003142 | 0.004094 | 0.004464 | 0.055292 | 0.704066 | 0.941582 |
| Faecalibacterium_prausnitzii | 1.403051 | 3.721905 | 3.571575 | 7.978344 | 0.767137 | 0.967639 |
| Flavobacterium_gelidilacus | 0 | 0 | 0 | 0 | 0.689157 | 0.941582 |
| Gemmiger_formicilis | 0.181009 | 0.75247 | 0.104631 | 0.227377 | 0.658541 | 0.941582 |
| Haemophilus_parainfluenzae | 0.002047 | 0.042421 | 0.015432 | 0.057382 | 0.394855 | 0.941582 |
| Kingella_potus | 0 | 0 | 0 | 0 | 0.39968 | 0.941582 |
| Kocuria_palustris | 0 | 0 | 0 | 0 | 0.057433 | 0.70834 |
| Lachnoanaerobaculum_orale | 0 | 0 | 0 | 0.000358 | 0.243428 | 0.941582 |
| Lactobacillus_delbrueckii | 0 | 0 | 0 | 0 | 0.348145 | 0.941582 |
| Lactobacillus_helveticus | 0 | 0 | 0 | 0 | 0.279016 | 0.941582 |
| Lactobacillus_mucosae | 0 | 0 | 0 | 0 | 0.673685 | 0.941582 |
| Lactobacillus_reuteri | 0 | 0 | 0 | 0.000307 | 1 | 1 |
| Lactobacillus_salivarius | 0 | 0.000974 | 0 | 0.02952 | 0.673149 | 0.941582 |
| Lactobacillus_zeae | 0 | 0 | 0 | 0 | 0.057433 | 0.70834 |
| Lactococcus_garvieae | 0 | 0 | 0 | 0 | 0.508149 | 0.941582 |
| Moryella_indoligenes | 0 | 0 | 0 | 0 | 0.689157 | 0.941582 |
| Neisseria_subflava | 0 | 0 | 0 | 0.002714 | 0.337707 | 0.941582 |
| Oryza_sativa_Indica_Group | 0 | 0 | 0 | 0 | 0.516003 | 0.941582 |
| Oscillospira_guilliermondii | 0 | 0 | 0 | 0.001056 | 0.820697 | 0.979542 |
| Oxalobacter_formigenes | 0 | 0.009679 | 0 | 0.005312 | 0.940377 | 0.990733 |
| Papillibacter_cinnamivorans | 0 | 0 | 0 | 0 | 0.39968 | 0.941582 |
| Parabacteroides_distasonis | 0.55305 | 1.304025 | 0.529933 | 0.954349 | 0.921476 | 0.990733 |
| Parabacteroides_gordonii | 0 | 0 | 0 | 0 | 0.516003 | 0.941582 |
| Paracoccus_aminovorans | 0 | 0 | 0 | 0 | 0.689157 | 0.941582 |
| Paracoccus_marcusii | 0 | 0 | 0 | 0 | 0.689157 | 0.941582 |
| Paraeggerthella  _hongkongensis | 0 | 0 | 0 | 0 | 0.057433 | 0.70834 |
| Prevotella_copri | 0.157584 | 32.470575 | 0.001038 | 0.066289 | 0.330965 | 0.941582 |
| Prevotella_intermedia | 0 | 0 | 0 | 0 | 0.689157 | 0.941582 |
| Prevotella_melaninogenica | 0 | 0 | 0 | 0 | 0.516003 | 0.941582 |
| Prevotella_nanceiensis | 0 | 0 | 0 | 0 | 0.689157 | 0.941582 |
| Prevotella_stercorea | 0 | 0.4974 | 0 | 0 | 0.389091 | 0.941582 |
| Prevotella_tannerae | 0 | 0 | 0 | 0 | 0.39968 | 0.941582 |
| Propionibacterium_acnes | 0 | 0 | 0 | 0 | 0.689157 | 0.941582 |
| Pseudomonas_stutzeri | 0 | 0 | 0 | 0 | 0.516003 | 0.941582 |
| Psychrobacter_pulmonis | 0 | 0 | 0 | 0 | 0.057433 | 0.70834 |
| Pyramidobacter_piscolens | 0 | 0 | 0 | 0 | 0.884073 | 0.990733 |
| Robinsoniella_peoriensis | 0 | 0 | 0 | 0 | 0.516003 | 0.941582 |
| Roseburia_faecis | 2.003237 | 2.020735 | 1.055247 | 3.292491 | 0.767137 | 0.967639 |
| Roseburia_inulinivorans | 0 | 0 | 0 | 0 | 0.39968 | 0.941582 |
| Rothia_dentocariosa | 0 | 0 | 0 | 0 | 0.508149 | 0.941582 |
| Rothia_mucilaginosa | 0 | 0 | 0 | 0.002088 | 0.452628 | 0.941582 |
| Ruminococcus_albus | 0 | 0 | 0 | 0 | 0.39968 | 0.941582 |
| Ruminococcus_bromii | 1.054252 | 0.845739 | 0.260884 | 2.20329 | 0.518347 | 0.941582 |
| Ruminococcus_callidus | 0.011615 | 0.130861 | 0.015765 | 0.090837 | 0.655293 | 0.941582 |
| Ruminococcus_gnavus | 0.405217 | 0.164358 | 0.173914 | 0.362081 | 0.148315 | 0.941582 |
| Ruminococcus_torques | 0.32242 | 0.425903 | 0.041741 | 0.128443 | 0.431526 | 0.941582 |
| Shinella_granuli | 0 | 0 | 0 | 0 | 0.689157 | 0.941582 |
| Sphingobacterium_mizutaii | 0 | 0 | 0 | 0 | 0.057433 | 0.70834 |
| Staphylococcus_succinus | 0 | 0 | 0 | 0 | 0.873197 | 0.990733 |
| Stenotrophomonas  _acidaminiphila | 0 | 0 | 0 | 0 | 0.057433 | 0.70834 |
| Streptococcus_anginosus | 0.032751 | 0.031114 | 0.002055 | 0.010144 | 0.097424 | 0.941582 |
| Streptococcus_infantis | 0.020469 | 0.009606 | 0.012297 | 0.063872 | 0.972882 | 0.990733 |
| Streptococcus_luteciae | 0 | 0 | 0 | 0.002175 | 0.285851 | 0.941582 |
| Streptococcus_sobrinus | 0 | 0 | 0 | 0 | 0.957573 | 0.990733 |
| Succinatimonas_hippei | 0 | 0 | 0 | 0 | 0.689157 | 0.941582 |
| Unclassified | 57.397552 | 20.402097 | 56.531987 | 17.051022 | 0.921476 | 0.990733 |
| Veillonella_dispar | 0.042985 | 0.068157 | 0.025738 | 0.143039 | 0.530435 | 0.941582 |
| Veillonella_parvula | 0.008518 | 0.064249 | 0.006619 | 0.015776 | 0.702028 | 0.941582 |
| Victivallis_vadensis | 0 | 0 | 0 | 0.003219 | 0.683297 | 0.941582 |
