# Supplementary material for: The Relationship Between Gut Microbiome Features and Chemotherapy Response in Gastrointestinal Cancer
Source: Front Oncol. 2021 Dec 23;11:781697. doi: 10.3389/fonc.2021.781697 (PMC8733568; doi:10.3389/fonc.2021.781697)
Supplement: Supplementary file 7 [file Table_3.doc]

**Supplemntal Table 3.** Baseline fecal microbiota between R and NR in gastric cancer (GC) group (n=43)

| **Species** | **Median**  **(NR.GC)** | **IQR**  **(NR.GC)** | **Median**  **(R.GC)** | **IQR**  **(R.GC)** | **p.value** | **FDR** |
| --- | --- | --- | --- | --- | --- | --- |
| Acinetobacter_guillouiae | 0 | 0 | 0 | 0 | 0.517113 | 0.886075 |
| Aggregatibacter_segnis | 0 | 0.002511 | 0 | 0.005095 | 0.96478 | 0.96478 |
| Akkermansia_muciniphila | 0.304383 | 0.785445 | 0.001717 | 0.242804 | 0.191935 | 0.841561 |
| Alistipes_finegoldii | 0.012791 | 0.014176 | 0.01008 | 0.039654 | 0.685174 | 0.904348 |
| Alistipes_indistinctus | 0.047943 | 0.112344 | 0.025167 | 0.135613 | 0.637352 | 0.886075 |
| Arcobacter_cryaerophilus | 0 | 0 | 0 | 0 | 0.021656 | 0.552626 |
| Bacteroides_caccae | 0.171278 | 0.398771 | 0.202292 | 0.637151 | 0.619448 | 0.886075 |
| Bacteroides_coprophilus | 0 | 0 | 0 | 0 | 0.833424 | 0.914717 |
| Bacteroides_eggerthii | 0.081859 | 0.305189 | 0.116116 | 0.539875 | 0.855648 | 0.914717 |
| Bacteroides_fragilis | 0.679516 | 1.572933 | 0.459871 | 1.299074 | 0.706026 | 0.904348 |
| Bacteroides_ovatus | 1.515828 | 1.408983 | 0.282299 | 0.818905 | 0.085908 | 0.810192 |
| Bacteroides_plebeius | 2.618671 | 4.803183 | 0.101496 | 3.247574 | 0.424094 | 0.886075 |
| Bacteroides_uniformis | 0.442688 | 0.220378 | 1.165249 | 3.124862 | 0.486239 | 0.886075 |
| Barnesiella_intestinihominis | 0 | 0 | 0 | 0 | 0.586214 | 0.886075 |
| Bifidobacterium_adolescentis | 0.00527 | 0.052698 | 0.015077 | 0.075303 | 0.698072 | 0.904348 |
| Blautia_obeum | 0.038109 | 0.033036 | 0.064768 | 0.096072 | 0.619448 | 0.886075 |
| Blautia_producta | 0.018263 | 0.092146 | 0.011148 | 0.040181 | 0.452447 | 0.886075 |
| Brevundimonas_vesicularis | 0 | 0 | 0 | 0 | 0.586214 | 0.886075 |
| Bulleidia_moorei | 0 | 0 | 0 | 0.001624 | 0.346868 | 0.886075 |
| Butyricicoccus_pullicaecorum | 0.051284 | 0.150296 | 0.04051 | 0.111677 | 0.348599 | 0.886075 |
| Butyrivibrio_crossotus | 0 | 0 | 0 | 0 | 0.292132 | 0.886075 |
| Capnocytophaga_ochracea | 0 | 0 | 0 | 0 | 0.586214 | 0.886075 |
| Cardiobacterium_valvarum | 0 | 0 | 0 | 0 | 0.833424 | 0.914717 |
| Clostridium_aldenense | 0.003837 | 0.008917 | 0.008148 | 0.043757 | 0.633801 | 0.886075 |
| Clostridium_celatum | 0 | 0.003166 | 0.010058 | 0.086172 | 0.052432 | 0.807451 |
| Clostridium_citroniae | 0.102324 | 0.166837 | 0.017265 | 0.084728 | 0.479174 | 0.886075 |
| Clostridium_clostridioforme | 0.092597 | 0.151115 | 0.1768 | 0.376126 | 0.706026 | 0.904348 |
| Clostridium_colicanis | 0 | 0 | 0 | 0 | 0.463633 | 0.886075 |
| Clostridium_colinum | 0 | 0 | 0 | 0.000623 | 0.350895 | 0.886075 |
| Clostridium_hathewayi | 0.03688 | 0.066917 | 0.031229 | 0.071 | 0.478953 | 0.886075 |
| Clostridium_hungatei | 0 | 0 | 0 | 0 | 0.903253 | 0.918627 |
| Clostridium_lavalense | 0.004054 | 0.004147 | 0.005602 | 0.020309 | 0.885379 | 0.918133 |
| Clostridium_methylpentosum | 0.002 | 0.002635 | 0.000624 | 0.00215 | 0.510474 | 0.886075 |
| Clostridium_paraputrificum | 0 | 0 | 0 | 0.001629 | 0.226491 | 0.860666 |
| Clostridium_perfringens | 0 | 0.004274 | 0 | 0.01326 | 0.910569 | 0.918627 |
| Clostridium_ramosum | 0 | 0.045586 | 0.000563 | 0.006965 | 0.878998 | 0.918133 |
| Clostridium_ruminantium | 0 | 0 | 0 | 0.009217 | 0.070829 | 0.807451 |
| Clostridium_sordellii | 0 | 0 | 0 | 0 | 0.85855 | 0.914717 |
| Clostridium_spiroforme | 0 | 0 | 0 | 0.00049 | 0.111304 | 0.810192 |
| Clostridium_symbiosum | 0 | 0.007376 | 0.002288 | 0.050159 | 0.263368 | 0.886075 |
| Collinsella_aerofaciens | 0.032468 | 0.02577 | 0.019015 | 0.028178 | 0.452447 | 0.886075 |
| Collinsella_stercoris | 0 | 0 | 0 | 0 | 0.15411 | 0.836597 |
| Coprococcus_catus | 0.020292 | 0.14811 | 0.029474 | 0.061996 | 0.370463 | 0.886075 |
| Coprococcus_eutactus | 0.100016 | 0.308686 | 0.004029 | 0.058099 | 0.273578 | 0.886075 |
| Corynebacterium_durum | 0 | 0 | 0 | 0 | 0.903253 | 0.918627 |
| Defluviitalea_saccharophila | 0.007533 | 0.010146 | 0.001048 | 0.006603 | 0.427015 | 0.886075 |
| Desulfovibrio_D168 | 0 | 0 | 0 | 0.542221 | 0.33577 | 0.886075 |
| Desulfurispirillum  _alkaliphilum | 0 | 0 | 0 | 0 | 0.586214 | 0.886075 |
| Dorea_formicigenerans | 0.040584 | 0.021931 | 0.027569 | 0.036548 | 0.188196 | 0.841561 |
| Eggerthella_lenta | 0.002029 | 0.002511 | 0 | 0.001618 | 0.176323 | 0.837534 |
| Enterococcus_casseliflavus | 0 | 0 | 0 | 0 | 0.292132 | 0.886075 |
| Escherichia_coli | 0.052728 | 0.573545 | 1.124251 | 5.492494 | 0.06519 | 0.807451 |
| Eubacterium_biforme | 0 | 0.387552 | 0 | 0.129655 | 0.655878 | 0.900844 |
| Eubacterium_cylindroides | 0 | 0 | 0 | 0 | 0.525324 | 0.886075 |
| Eubacterium_dolichum | 0.001318 | 0.007533 | 0.002963 | 0.023482 | 0.818549 | 0.914717 |
| Faecalibacterium_prausnitzii | 2.122598 | 3.2068 | 1.424221 | 2.835268 | 0.222025 | 0.860666 |
| Flavobacterium_gelidilacus | 0 | 0 | 0 | 0 | 0.117594 | 0.810192 |
| Flavobacterium_succinicans | 0 | 0 | 0 | 0 | 0.406203 | 0.886075 |
| Gemmiger_formicilis | 0.174563 | 0.552715 | 0.039608 | 0.228842 | 0.215457 | 0.860666 |
| Haemophilus_parainfluenzae | 0.088648 | 0.269544 | 0.170378 | 0.486043 | 0.735788 | 0.914717 |
| Kingella_potus | 0 | 0 | 0 | 0 | 0.586214 | 0.886075 |
| Lachnoanaerobaculum_orale | 0 | 0.001279 | 0 | 0.001326 | 0.699939 | 0.904348 |
| Lactobacillus_delbrueckii | 0 | 0 | 0 | 0 | 0.021656 | 0.552626 |
| Lactobacillus_helveticus | 0.002511 | 0.006001 | 0 | 0 | 0.008058 | 0.552626 |
| Lactobacillus_mucosae | 0 | 0.016003 | 0 | 0 | 0.024238 | 0.552626 |
| Lactobacillus_reuteri | 0.001425 | 0.04459 | 0 | 0.002466 | 0.060207 | 0.807451 |
| Lactobacillus_salivarius | 0.017095 | 0.493058 | 0 | 0.07882 | 0.037308 | 0.708852 |
| Lactobacillus_zeae | 0 | 0 | 0 | 0 | 0.609521 | 0.886075 |
| Lactococcus_garvieae | 0 | 0 | 0 | 0 | 0.48955 | 0.886075 |
| Malus_x_domestica | 0 | 0 | 0 | 0 | 0.117594 | 0.810192 |
| Morganella_morganii | 0 | 0 | 0 | 0 | 0.586214 | 0.886075 |
| Neisseria_subflava | 0 | 0.001255 | 0.00136 | 0.003713 | 0.472942 | 0.886075 |
| Olsenella_umbonata | 0 | 0 | 0 | 0 | 0.839457 | 0.914717 |
| Oryza_sativa_Indica_Group | 0 | 0 | 0 | 0 | 0.15411 | 0.836597 |
| Oscillospira_guilliermondii | 0 | 0.008117 | 0 | 0 | 0.164228 | 0.837534 |
| Oxalobacter_formigenes | 0.002635 | 0.056494 | 0.006078 | 0.017176 | 0.852962 | 0.914717 |
| Papillibacter_cinnamivorans | 0 | 0 | 0 | 0 | 0.627073 | 0.886075 |
| Parabacteroides_distasonis | 0.801515 | 0.700205 | 0.65927 | 1.96511 | 0.858289 | 0.914717 |
| Parabacteroides_gordonii | 0 | 0 | 0 | 0 | 0.721623 | 0.914056 |
| Paracoccus_aminovorans | 0 | 0 | 0 | 0 | 0.463633 | 0.886075 |
| Paracoccus_marcusii | 0 | 0 | 0 | 0 | 0.525324 | 0.886075 |
| Porphyromonas_endodontalis | 0 | 0 | 0 | 0 | 0.586214 | 0.886075 |
| Prevotella_copri | 0.001425 | 25.430703 | 0.004098 | 13.865971 | 0.854207 | 0.914717 |
| Prevotella_intermedia | 0 | 0 | 0 | 0 | 0.406203 | 0.886075 |
| Prevotella_melaninogenica | 0 | 0.001229 | 0 | 0 | 0.127925 | 0.810192 |
| Prevotella_stercorea | 0 | 0.184183 | 0 | 0.175896 | 0.622353 | 0.886075 |
| Prevotella_tannerae | 0 | 0 | 0 | 0 | 0.123642 | 0.810192 |
| Pseudomonas_stutzeri | 0 | 0 | 0 | 0 | 0.7767 | 0.914717 |
| Psychrobacter_pulmonis | 0 | 0 | 0 | 0 | 0.021656 | 0.552626 |
| Pyramidobacter_piscolens | 0 | 0.008117 | 0 | 0.017055 | 0.760448 | 0.914717 |
| Robinsoniella_peoriensis | 0 | 0 | 0 | 0 | 0.525324 | 0.886075 |
| Roseburia_faecis | 1.611972 | 1.8486 | 0.311397 | 2.428762 | 0.706026 | 0.904348 |
| Roseburia_inulinivorans | 0 | 0 | 0 | 0 | 0.463633 | 0.886075 |
| Rothia_dentocariosa | 0 | 0 | 0 | 0 | 0.48955 | 0.886075 |
| Rothia_mucilaginosa | 0 | 0.001255 | 0.001966 | 0.003786 | 0.150336 | 0.836597 |
| Ruminococcus_albus | 0 | 0 | 0 | 0 | 0.586214 | 0.886075 |
| Ruminococcus_bromii | 0.073759 | 0.414696 | 0.488395 | 1.590455 | 0.089287 | 0.810192 |
| Ruminococcus_callidus | 0.016003 | 0.042824 | 0.014714 | 0.066061 | 0.885918 | 0.918133 |
| Ruminococcus_flavefaciens | 0 | 0 | 0 | 0 | 0.525324 | 0.886075 |
| Ruminococcus_gnavus | 0.118019 | 0.487125 | 0.212879 | 0.287837 | 0.619448 | 0.886075 |
| Ruminococcus_torques | 0.047431 | 0.139983 | 0.024663 | 0.060792 | 0.202152 | 0.853531 |
| Shuttleworthia_satelles | 0 | 0 | 0 | 0 | 0.117594 | 0.810192 |
| Sphingobacterium_mizutaii | 0 | 0 | 0 | 0 | 0.525324 | 0.886075 |
| Staphylococcus_succinus | 0 | 0 | 0 | 0 | 0.839457 | 0.914717 |
| Stenotrophomonas  _acidaminiphila | 0 | 0 | 0 | 0 | 0.525324 | 0.886075 |
| Streptococcus_anginosus | 0.003837 | 0.011239 | 0.003916 | 0.035723 | 0.807033 | 0.914717 |
| Streptococcus_infantis | 0.026364 | 0.095796 | 0.064191 | 0.141158 | 0.765939 | 0.914717 |
| Streptococcus_luteciae | 0.001318 | 0.002511 | 0.001956 | 0.0029 | 0.784589 | 0.914717 |
| Streptococcus_sobrinus | 0 | 0.006088 | 0 | 0.001982 | 0.247596 | 0.886075 |
| Unclassified | 67.051247 | 34.407732 | 55.864854 | 28.082493 | 0.511633 | 0.886075 |
| Veillonella_dispar | 0.174028 | 0.283913 | 0.101917 | 0.381671 | 0.808349 | 0.914717 |
| Veillonella_parvula | 0.002511 | 0.020579 | 0.020408 | 0.113137 | 0.17366 | 0.837534 |
| Vibrio_cholerae | 0 | 0 | 0 | 0 | 0.586214 | 0.886075 |
| Victivallis_vadensis | 0 | 0.010146 | 0 | 0.001948 | 0.311691 | 0.886075 |
