# Supplementary material for: The Relationship Between Gut Microbiome Features and Chemotherapy Response in Gastrointestinal Cancer
Source: Front Oncol. 2021 Dec 23;11:781697. doi: 10.3389/fonc.2021.781697 (PMC8733568; doi:10.3389/fonc.2021.781697)
Supplement: Supplementary file 8 [file Table_4.doc]

**Supplemntal Table 4.** Baseline fecal microbiota between R and NR in colorectal cancer (CRC) group (n=41)

| **Species** | **Median**  **(NR.CRC)** | **IQR**  **(NR.CRC)** | **Median**  **(R.CRC)** | **IQR**  **(R.CRC)** | **p.value** | **FDR** |
| --- | --- | --- | --- | --- | --- | --- |
| Acinetobacter_guillouiae | 0 | 0.00367 | 0 | 0.001671 | 0.902565 | 1 |
| Aggregatibacter_segnis | 0 | 0.002967 | 0 | 0.007482 | 0.474173 | 0.94561 |
| Akkermansia_muciniphila | 0.002216 | 1.196757 | 0.021366 | 2.809614 | 0.459891 | 0.94561 |
| Alistipes_finegoldii | 0.014013 | 0.020786 | 0.01491 | 0.047652 | 0.954421 | 1 |
| Alistipes_indistinctus | 0.05845 | 0.091825 | 0.037809 | 0.15184 | 0.784004 | 0.971475 |
| Arcobacter_cryaerophilus | 0 | 0 | 0 | 0 | 0.310853 | 0.94561 |
| Atopobium_vaginae | 0 | 0 | 0 | 0 | 0.190075 | 0.94561 |
| Bacteroides_caccae | 0.219658 | 0.422101 | 0.12832 | 0.734834 | 0.690895 | 0.94561 |
| Bacteroides_coprophilus | 0 | 0 | 0 | 0 | 0.862878 | 1 |
| Bacteroides_eggerthii | 0.184432 | 0.375103 | 0.108013 | 0.464129 | 0.660224 | 0.94561 |
| Bacteroides_fragilis | 1.232731 | 3.2022 | 1.061732 | 2.763622 | 0.899757 | 1 |
| Bacteroides_nordii | 0 | 0 | 0 | 0 | 0.495628 | 0.94561 |
| Bacteroides_ovatus | 1.719451 | 5.477733 | 1.232062 | 4.492969 | 0.63981 | 0.94561 |
| Bacteroides_plebeius | 0.000978 | 0.028254 | 0.000782 | 0.090852 | 0.903501 | 1 |
| Bacteroides_uniformis | 0.889106 | 1.354972 | 0.48298 | 3.015291 | 0.9547 | 1 |
| Barnesiella_intestinihominis | 0 | 0 | 0 | 0 | 0.495628 | 0.94561 |
| Bifidobacterium_adolescentis | 0.014688 | 0.08753 | 0.003008 | 0.045458 | 0.677795 | 0.94561 |
| Blautia_obeum | 0.011 | 0.022351 | 0.024112 | 0.064997 | 0.484758 | 0.94561 |
| Blautia_producta | 0.032595 | 0.1031 | 0.01033 | 0.042763 | 0.059273 | 0.94561 |
| Brachybacterium_conglomeratum | 0 | 0 | 0 | 0 | 0.310853 | 0.94561 |
| Brevundimonas_vesicularis | 0 | 0 | 0 | 0 | 0.748399 | 0.961179 |
| Bulleidia_moorei | 0 | 0.001121 | 0.001264 | 0.00446 | 0.203279 | 0.94561 |
| Butyricicoccus_pullicaecorum | 0.061151 | 0.145393 | 0.073088 | 0.151641 | 0.541899 | 0.94561 |
| Butyrivibrio_crossotus | 0 | 0 | 0 | 0 | 0.474286 | 0.94561 |
| Campylobacter_ureolyticus | 0 | 0 | 0 | 0.001488 | 0.362142 | 0.94561 |
| Cardiobacterium_valvarum | 0 | 0 | 0 | 0 | 0.203138 | 0.94561 |
| Clostridium_aldenense | 0.019981 | 0.03811 | 0.011336 | 0.066594 | 0.842404 | 1 |
| Clostridium_bifermentans | 0 | 0 | 0 | 0 | 0.495628 | 0.94561 |
| Clostridium_butyricum | 0 | 0 | 0 | 0 | 0.310853 | 0.94561 |
| Clostridium_celatum | 0.002887 | 0.011246 | 0.022321 | 0.121719 | 0.048954 | 0.94561 |
| Clostridium_cellulolyticum | 0 | 0 | 0 | 0 | 0.495628 | 0.94561 |
| Clostridium_citroniae | 0.235832 | 0.7656 | 0.041274 | 0.229126 | 0.122176 | 0.94561 |
| Clostridium_clostridioforme | 0.079389 | 1.324939 | 0.137019 | 0.433168 | 0.887069 | 1 |
| Clostridium_colicanis | 0 | 0 | 0 | 0 | 0.495628 | 0.94561 |
| Clostridium_colinum | 0 | 0.023868 | 0 | 0 | 0.227536 | 0.94561 |
| Clostridium_hathewayi | 0.095931 | 0.269472 | 0.055586 | 0.093855 | 0.16035 | 0.94561 |
| Clostridium_hungatei | 0 | 0 | 0 | 0 | 0.310853 | 0.94561 |
| Clostridium_lavalense | 0.009637 | 0.029013 | 0.024193 | 0.061555 | 0.504774 | 0.94561 |
| Clostridium_methylpentosum | 0.000816 | 0.009653 | 0.001978 | 0.005842 | 0.779227 | 0.971475 |
| Clostridium_neonatale | 0 | 0 | 0 | 0 | 0.567437 | 0.94561 |
| Clostridium_paraputrificum | 0 | 0 | 0 | 0.002304 | 0.358118 | 0.94561 |
| Clostridium_perfringens | 0 | 0 | 0 | 0.004903 | 0.162032 | 0.94561 |
| Clostridium_ramosum | 0.008653 | 0.027679 | 0.004011 | 0.107984 | 0.64286 | 0.94561 |
| Clostridium_ruminantium | 0 | 0 | 0 | 0.025057 | 0.00915 | 0.619433 |
| Clostridium_sordellii | 0 | 0 | 0 | 0 | 0.44435 | 0.94561 |
| Clostridium_spiroforme | 0 | 0 | 0 | 0.003598 | 0.421115 | 0.94561 |
| Clostridium_symbiosum | 0.021215 | 0.030847 | 0.012656 | 0.097575 | 0.842044 | 1 |
| Collinsella_aerofaciens | 0 | 0.009336 | 0.019415 | 0.056126 | 0.129696 | 0.94561 |
| Collinsella_stercoris | 0 | 0 | 0 | 0 | 0.652411 | 0.94561 |
| Coprococcus_catus | 0.020866 | 0.055243 | 0.033373 | 0.073448 | 0.760895 | 0.96774 |
| Coprococcus_eutactus | 0 | 0 | 0 | 0.033879 | 0.575723 | 0.94561 |
| Corynebacterium_durum | 0 | 0 | 0 | 0 | 0.310853 | 0.94561 |
| Defluviitalea_saccharophila | 0.00708 | 0.030775 | 0.003783 | 0.013575 | 0.589931 | 0.94561 |
| Desulfovibrio_D168 | 0 | 0 | 0 | 0.003245 | 0.304126 | 0.94561 |
| Dorea_formicigenerans | 0.015797 | 0.047875 | 0.03165 | 0.074726 | 0.241314 | 0.94561 |
| Eggerthella_lenta | 0.001623 | 0.00595 | 0 | 0.001494 | 0.066935 | 0.94561 |
| Elizabethkingia_meningoseptica | 0 | 0 | 0 | 0 | 0.652411 | 0.94561 |
| Enterococcus_casseliflavus | 0 | 0 | 0 | 0 | 0.310853 | 0.94561 |
| Escherichia_coli | 0.429126 | 3.164987 | 0.546655 | 3.503995 | 0.899757 | 1 |
| Eubacterium_biforme | 0 | 0 | 0 | 0 | 0.177449 | 0.94561 |
| Eubacterium_dolichum | 0.012414 | 0.072337 | 0.004731 | 0.055181 | 0.416368 | 0.94561 |
| Faecalibacterium_prausnitzii | 2.263455 | 5.142046 | 0.966569 | 2.542515 | 0.452304 | 0.94561 |
| Flavobacterium_gelidilacus | 0 | 0 | 0 | 0 | 0.624035 | 0.94561 |
| Flavobacterium_succinicans | 0 | 0 | 0 | 0 | 0.652411 | 0.94561 |
| Gemmiger_formicilis | 0.007214 | 0.026375 | 0.024359 | 0.16816 | 0.358803 | 0.94561 |
| Haemophilus_parainfluenzae | 0.004007 | 0.012002 | 0.005812 | 0.024042 | 0.694114 | 0.94561 |
| Kingella_potus | 0 | 0 | 0 | 0 | 0.310853 | 0.94561 |
| Kocuria_palustris | 0 | 0 | 0 | 0 | 0.663069 | 0.94561 |
| Lachnoanaerobaculum_orale | 0 | 0 | 0 | 0.001084 | 0.286241 | 0.94561 |
| Lactobacillus_delbrueckii | 0 | 0 | 0 | 0 | 0.495628 | 0.94561 |
| Lactobacillus_helveticus | 0 | 0.001778 | 0 | 0 | 0.31883 | 0.94561 |
| Lactobacillus_iners | 0 | 0 | 0 | 0 | 1 | 1 |
| Lactobacillus_mucosae | 0 | 0 | 0 | 0 | 0.592526 | 0.94561 |
| Lactobacillus_paralimentarius | 0 | 0 | 0 | 0 | 0.190075 | 0.94561 |
| Lactobacillus_reuteri | 0 | 0 | 0 | 0 | 0.44435 | 0.94561 |
| Lactobacillus_salivarius | 0 | 0 | 0 | 0.002118 | 0.484206 | 0.94561 |
| Lactobacillus_zeae | 0 | 0 | 0 | 0 | 1 | 1 |
| Lactococcus_garvieae | 0 | 0 | 0 | 0 | 0.684858 | 0.94561 |
| Malus_x_domestica | 0 | 0 | 0 | 0 | 0.786079 | 0.971475 |
| Massilia_haematophila | 0 | 0 | 0 | 0 | 0.707403 | 0.94561 |
| Morganella_morganii | 0 | 0 | 0 | 0 | 0.604898 | 0.94561 |
| Moryella_indoligenes | 0 | 0 | 0 | 0 | 0.310853 | 0.94561 |
| Neisseria_subflava | 0 | 0.003266 | 0.001958 | 0.004512 | 0.225359 | 0.94561 |
| Olsenella_umbonata | 0 | 0 | 0 | 0 | 0.495628 | 0.94561 |
| Oryza_sativa_Indica_Group | 0 | 0 | 0 | 0 | 0.707403 | 0.94561 |
| Oscillospira_guilliermondii | 0 | 0.002082 | 0 | 0 | 0.10451 | 0.94561 |
| Oxalobacter_formigenes | 0.011624 | 0.053204 | 0.006784 | 0.036333 | 0.988228 | 1 |
| Papillibacter_cinnamivorans | 0 | 0 | 0 | 0 | 0.862878 | 1 |
| Parabacteroides_distasonis | 0.678204 | 1.286665 | 0.540901 | 1.716117 | 0.966041 | 1 |
| Parabacteroides_gordonii | 0 | 0 | 0 | 0 | 0.967627 | 1 |
| Paracoccus_aminovorans | 0 | 0 | 0 | 0 | 0.824268 | 1 |
| Paracoccus_marcusii | 0 | 0 | 0 | 0 | 0.975237 | 1 |
| Paraeggerthella_hongkongensis | 0 | 0 | 0 | 0 | 1 | 1 |
| Porphyromonas_endodontalis | 0 | 0 | 0 | 0.001174 | 0.11347 | 0.94561 |
| Prevotella_copri | 0 | 0.026903 | 0.005837 | 0.027991 | 0.298166 | 0.94561 |
| Prevotella_intermedia | 0 | 0.037714 | 0 | 0.040359 | 0.946918 | 1 |
| Prevotella_melaninogenica | 0 | 0 | 0 | 0 | 0.624035 | 0.94561 |
| Prevotella_nanceiensis | 0 | 0 | 0 | 0 | 0.495628 | 0.94561 |
| Prevotella_nigrescens | 0 | 0 | 0 | 0 | 0.925807 | 1 |
| Prevotella_stercorea | 0 | 0 | 0 | 0.000807 | 0.362142 | 0.94561 |
| Prevotella_tannerae | 0 | 0 | 0 | 0.001174 | 0.654553 | 0.94561 |
| Propionibacterium_acnes | 0 | 0 | 0 | 0 | 0.310853 | 0.94561 |
| Pseudomonas_stutzeri | 0 | 0 | 0 | 0 | 0.266873 | 0.94561 |
| Psychrobacter_pulmonis | 0 | 0 | 0 | 0 | 0.624035 | 0.94561 |
| Pyramidobacter_piscolens | 0.002933 | 0.016787 | 0 | 0.005344 | 0.475095 | 0.94561 |
| Robinsoniella_peoriensis | 0 | 0 | 0 | 0 | 0.462212 | 0.94561 |
| Roseburia_faecis | 0.04613 | 0.843646 | 0.360468 | 1.790294 | 0.273695 | 0.94561 |
| Roseburia_inulinivorans | 0 | 0 | 0 | 0 | 0.203138 | 0.94561 |
| Rothia_dentocariosa | 0 | 0 | 0 | 0 | 0.624035 | 0.94561 |
| Rothia_mucilaginosa | 0 | 0.000745 | 0.000723 | 0.002137 | 0.142934 | 0.94561 |
| Ruminococcus_albus | 0 | 0 | 0 | 0 | 0.310853 | 0.94561 |
| Ruminococcus_bromii | 0 | 0.921655 | 0.223077 | 1.336387 | 0.337553 | 0.94561 |
| Ruminococcus_callidus | 0 | 0 | 0.005182 | 0.045382 | 0.111617 | 0.94561 |
| Ruminococcus_flavefaciens | 0 | 0 | 0 | 0 | 0.925807 | 1 |
| Ruminococcus_gnavus | 0.169273 | 0.25794 | 0.162449 | 0.247568 | 0.584707 | 0.94561 |
| Ruminococcus_torques | 0.152513 | 0.284345 | 0.104265 | 0.242389 | 0.74412 | 0.961179 |
| Shinella_granuli | 0 | 0 | 0 | 0 | 0.925807 | 1 |
| Shuttleworthia_satelles | 0 | 0 | 0 | 0 | 0.925807 | 1 |
| Sphingobacterium_mizutaii | 0 | 0 | 0 | 0 | 0.624035 | 0.94561 |
| Staphylococcus_succinus | 0 | 0 | 0 | 0 | 0.743849 | 0.961179 |
| Stenotrophomonas_acidaminiphila | 0 | 0 | 0 | 0 | 0.495628 | 0.94561 |
| Streptococcus_anginosus | 0.002051 | 0.005457 | 0.000969 | 0.007354 | 0.626341 | 0.94561 |
| Streptococcus_infantis | 0.003934 | 0.003743 | 0.015123 | 0.036419 | 0.009457 | 0.619433 |
| Streptococcus_luteciae | 0 | 0 | 0 | 0 | 0.611942 | 0.94561 |
| Streptococcus_sobrinus | 0 | 0 | 0 | 0 | 0.925807 | 1 |
| Treponema_amylovorum | 0 | 0 | 0 | 0 | 0.310853 | 0.94561 |
| Treponema_socranskii | 0 | 0 | 0 | 0 | 0.743849 | 0.961179 |
| Unclassified | 69.39429 | 31.438185 | 57.59573 | 18.77517 | 0.705348 | 0.94561 |
| Veillonella_dispar | 0.005701 | 0.007114 | 0.00722 | 0.056427 | 0.449826 | 0.94561 |
| Veillonella_parvula | 0 | 0.001267 | 0 | 0.003912 | 0.403794 | 0.94561 |
| Victivallis_vadensis | 0 | 0 | 0 | 0.008021 | 0.125623 | 0.94561 |
